# Association of Inflammatory Cytokines With Non-Alcoholic Fatty Liver Disease

**DOI:** 10.3389/fimmu.2022.880298

**Published:** 2022-05-06

**Authors:** Yamei Duan, Xiongfeng Pan, Jiayou Luo, Xiang Xiao, Jingya Li, Prince L. Bestman, Miyang Luo

**Affiliations:** ^1^ Department of Maternal and Child Health, Xiangya School of Public Health, Central South University, Changsha, China; ^2^ Department of Epidemiology and Health Statistics, Xiangya School of Public Health, Central South University, Changsha, China

**Keywords:** inflammatory cytokines, non-alcoholic fatty liver disease, non-alcoholic steatohepatitis, hepatic steatosis, hepatic fibrosis

## Abstract

**Background:**

Inflammatory cytokines have been considered to be significant factors contributing to the development and progression of non-alcoholic fatty liver disease (NAFLD). However, the role of inflammatory cytokines in NAFLD remains inconclusive.

**Objective:**

This study aimed to evaluate the association between inflammatory cytokines and NAFLD.

**Methods:**

PubMed, Web of Science, the Cochrane Library, and EMBASE databases were searched until 31 December 2021 to identify eligible studies that reported the association of inflammatory cytokine with NAFLD and its subtypes. We pooled odds ratios (ORs) and hazard risk (HRs) with 95% confidence intervals (CIs) and conducted heterogeneity tests. Sensitivity analysis and analysis for publication bias were also carried out.

**Results:**

The search in the databases identified 51 relevant studies that investigated the association between 19 different inflammatory cytokines and NAFLD based on 36,074 patients and 47,052 controls. The results of the meta-analysis showed significant associations for C-reactive protein (CRP), interleukin-1β (IL-1β), interleukin-6 (IL-6), tumor necrosis factor-α (TNF-α), and intercellular adhesion molecule-1 (ICAM-1) with NAFLD (ORs of 1.41, 1.08, 1.50, 1.15 and 2.17, respectively). In contrast, we observed non-significant associations for interferon-γ (IFN-γ), insulin-like growth factor (IGF-II), interleukin-2 (IL-2), interleukin-4 (IL-4), interleukin-5 (IL-5), interleukin-7 (IL-7), interleukin-8 (IL-8), interleukin-10 (IL-10), interleukin-12 (IL-12), monocyte chemoattractant protein-1(MCP-1), and transforming growth factor-β (TGF-β) with NAFLD. Our results also showed that CRP, IL-1β, and TNF-α were significantly associated with non-alcoholic steatohepatitis (NASH) and hepatic fibrosis.

**Conclusions:**

Our results indicated that increased CRP, IL‐1β, IL-6, TNF‐α, and ICAM-1 concentrations were significantly associated with increased risks of NAFLD. These inflammatory mediators may serve as biomarkers for NAFLD subjects and expect to provide new insights into the aetiology of NAFLD as well as early diagnosis and intervention.

## Introduction

Non-alcoholic fatty liver disease (NAFLD) has become the most common chronic liver disease worldwide, with a global prevalence of 25% in the general population (1), and 80% in the obese population (2). NAFLD is well recognized as a hepatic manifestation of metabolic syndrome, with strong links to obesity, insulin resistance, increased systemic inflammation, and advanced atherosclerosis (3).

The pathogenesis of NAFLD has not been fully elucidated. The mechanism of progression is usually explained by the classic “multiple strikes” theory of NAFLD pathogenesis, which states that lipid accumulation triggers hepatic steatosis, leading to multiple injuries, including adipokine secretion, inflammation, lipotoxicity, and dysregulation of glucose and lipid metabolism, which may eventually cause non-alcoholic steatohepatitis (NASH) and cirrhosis (4–6).

It is widely accepted that cytokines play a critical role as mediators of inflammation, fibrosis, and cirrhosis in NAFLD (7). Previous studies have reported several inflammatory mediators involved in the development and progression of NAFLD, such as interleukin-1β (IL-1β), interleukin-6 (IL-6), tumor necrosis factor-α (TNF-α), C-reactive protein (CRP) and NOD-like receptor protein 3 (NLRP3) inflammasome (8–10). Some of these inflammatory mediators with immunomodulatory functions can be used as biomarkers to assess the severity and predict the prognosis of NAFLD (8). However, previous studies reported an inconsistent association between inflammatory cytokines and NAFLD. Some studies found positive associations between inflammatory cytokines and NAFLD (11, 12), while other studies found negative or null associations (13, 14).

Several studies have reviewed inflammatory mediators and molecular pathways involved in the development and progression of NAFLD (15–18). However, to our knowledge, few meta-analyses have comprehensively assessed the association between inflammatory cytokines and NAFLD. For instance, one previous review summarized the beneficial effects and detrimental effects of inflammatory mediators and inflammatory cells in NAFLD (18). However, this review was restricted to six inflammatory cytokines and did not review the role of other inflammatory cytokines in NAFLD. In addition, there was no quantification of the effect of inflammatory cytokines on NAFLD. In recent years, a growing number of studies have been conducted in various countries to analyze this topic (19), thus it is essential to conduct a meta-analysis to assess the association between inflammatory cytokines and NAFLD.

The aim of this study was to provide a complete review of existing cross-sectional studies, case-control studies, and cohort studies on the association between inflammatory cytokines and NAFLD. Furthermore, we classified NAFLD into different types by disease spectrum (e.g., NASH and hepatic steatosis), and performed subgroup analyses to elucidate the essential association between inflammatory cytokines and NAFLD.

## Methods

This review and meta‐analysis were conducted in accordance with the Preferred Reporting Items for Systematic Reviews and Meta‐Analyses (PRISMA) (20).

### Search Strategy

Potentially relevant articles were searched using electronic databases. We systematically searched PubMed, Web of Science, the Cochrane Library, and EMBASE databases for articles published from 1 January 1960 to 31 December 2021. The search strategy included all possible combinations of keywords from the three groups related to non-alcoholic fatty liver disease, inflammatory cytokines (including interleukin, interferon, tumor necrosis factor, growth factor, and chemokine), and their associated outcomes. The specific search strategy is provided in **Supplementary Table 1**.

### Selection Criteria

Studies that met all of the following criteria were included in the study: (a) the patients in the studies were diagnosed with NAFLD, including hepatic steatosis, NASH, and hepatic fibrosis; (b) observational studies (cross-sectional studies, case-control studies, and cohort studies) that reported the estimates of the effect of inflammatory cytokines on NAFLD, including odds ratios (ORs), relative risks (RRs) or hazard ratios (HRs), and 95% confidence intervals (CIs); (c) study subjects were human populations; and (d) articles were written in English.

Studies that met any of the following criteria were excluded from the study: (a) only conducted *in vivo* or *in vitro* cell experiments; (b) with incomplete data in the original studies; (c) duplicated studies retrieved from various databases; and (d) reviews, meta-analyses, clinical guidelines, comments, letters to the editor, or case reports.

### Data Extraction

A standardized data extraction form was used to extract data from each eligible study by two reviewers (YD and XP) independently. Any further inconsistencies were addressed by a joint discussion. The following data were recorded: name of the first author, publication year, country, sample size, type of case/control, mean age (years), BMI (kg/m^2^), sample type, subtypes of NAFLD, the effect of inflammatory cytokines on the ORs/RRs/HRs and 95% CIs of NAFLD, diagnostic methods for NAFLD, and measurements of inflammatory cytokines.

### Quality Assessment

The methodological quality of the eligible studies was assessed using the National Institutes of Health’s Quality Assessment Tool for Observational Cohort and Cross‐Sectional Studies (NIHQAT) (21).This assessment tool rates each study based on 14 criteria (**Supplementary Table 2**). A study‐specific global score ranging from zero to 14 was calculated by summing up scores across all criteria. The study quality assessment helped measure the strength of scientific evidence but was not used to determine the inclusion of studies.

### Statistical Analysis

Each inflammatory cytokine with more than two identified studies was included in the meta-analysis. The ORs with 95% CIs were used to describe the effect of inflammatory cytokines on NAFLD. The heterogeneity was evaluated by I-squared (*I^2^*). The level of heterogeneity measured by *I^2^* was interpreted as modest (*I^2^* ≤ 25%), moderate (25%< *I^2^* ≤50%), substantial (50% < *I^2^* ≤ 75%), or considerable (*I^2^* > 75%). Q-statistic tests were also conducted, where *P* < 0.1 indicates the presence of heterogeneity among studies. If *I^2^* ≥ 50% or *P* < 0.1, the random effect model was used, otherwise the fixed effect model was applied. Subgroup analyses were used to explore the potential sources of heterogeneity. Sensitivity analyses were performed to evaluate the availability and reliability of the results by deleting one study at a time and combining the effect values of the remaining studies. Funnel plots with Begg’s tests and Egger’s tests were used to assess publication bias. All statistical tests were two-sided; *P* < 0.05 was considered statistically significant except for the evaluation of heterogeneity (*P* < 0.1). All meta‐analyses were performed using Stata v12.0 (StataCorp, College Station, TX, USA).

## Results

### Study Selection

**Figure 1:**
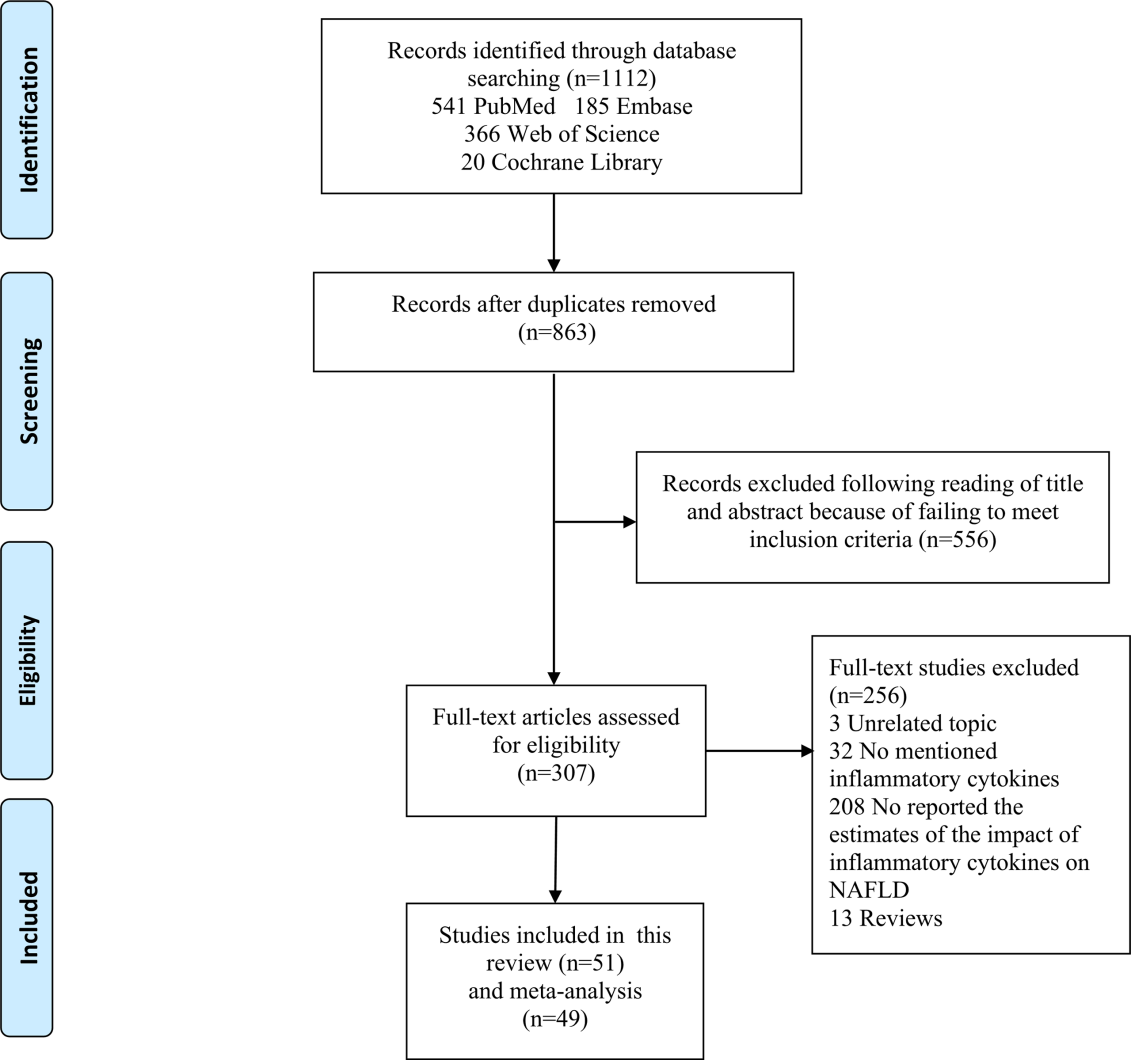
Flowchart of study inclusion and exclusion.

**Figure 1** shows the flow chart of study selection. The search strategy identified a total of 1,112 articles in the databases and 863 studies remained after excluding duplicated articles. Title and abstract screening excluded 556 articles. Full texts of the remaining 307 articles were reviewed according to the selection criteria, and this process excluded 256 articles. The remaining 51 studies (22–72) were included in this study, and 49 of them were included in the meta‐analyses.

### Study Characteristics

Characteristics of the 51 eligible studies are summarized in **Table 1** and **Supplementary Table 3–5**. All the studies were published between 1960 and 2021, with 32 cross‐sectional studies, eight case‐control studies, and 11 cohort studies. A total of 36,074 NAFLD patients and 47,052 controls were included in these studies. Of these 51 studies, 49 studied on CRP, six on IL‐1β, five on interleukin-2 (IL-2), four on interleukin-4 (IL-4), four on interleukin-5 (IL-5), 12 on IL‐6, four on interleukin-7 (IL-7), four on interleukin-8 (IL-8), four on interleukin-10 (IL-10), three on interleukin-12 (IL-12), 11 on TNF-α, 10 on transforming growth factor-β (TGF-β), six on monocyte chemoattractant protein-1(MCP-1), four on interferon-γ (IFN-γ), four on insulin-like growth factor (IGF-II), and four on intercellular adhesion molecule-1 (ICAM-1). Sources of inflammatory cytokines included serum samples in 28 studies, plasma samples in 11 studies, and whole blood samples in six studies, while the source of inflammatory cytokines in six studies was unclear. 22 of these studies used an enzyme‐linked immunosorbent assay (ELISA) to detect the concentrations of inflammatory cytokines, and 29 studies used other methods, such as Luminex Multiplex, Cytokine Antibody Array, Turbidimetric inhibition immunoassay, and Latex agglutination assay. For the quality score, 51 studies scored between 8 and 14 with an average score of 9.45.

**Table 1:** Characteristics of included studies.

Study (Year)	Reference	Design type	No. of cases	No. of controls	Case type	Inflammatory cytokines	Study quality
Abdel-Razik (2016)	(22)	Cohort	120	NR	Hepatic fibrosis	TNF-α/IL-6	9
Ajmera (2017)	(23)	Cross-sectional	376/288	272/360	NASH	IFN-γ/IGF-II/IL-1β/IL-2/IL-4/IL-5/IL-6/IL-7/IL-8/IL-10/IL-12/MCP-1/TGF-β/TNF-α	9
Akinkugbe (2017)	(24)	Cross-sectional	654	1827	NAFLD	CRP	8
Alisi (2010)	(25)	Cross-sectional	40	9	NASH	TNF-α/IL-6	9
Alrifai (2015)	(26)	Cross-sectional	670	3306	NAFLD	CRP	9
Barretto (2020)	(27)	Cross-sectional	36	36	Hepatic steatosis	TGF-β	9
Ceccarelli (2015)	(28)	Cohort	40	8	NASH/Hepatic fibrosis	IL-1β	12
Chiang (2012)	(29)	Case-control	34	68	NAFLD	CRP	9
Choi (2009)	(30)	Cross-sectional	5769	11581	NAFLD	CRP	9
Chunming (2015)	(31)	Cross-sectional	28	54	NAFLD	CRP	9
El-Ashmawy (2019)	(32)	Cross-sectional	93	72	NAFLD	CRP	9
García-Galiano (2007)	(33)	Cross-sectional	9	27	Hepatic steatosis	IGF-I/IL-6	8
Holterman (2013)	(34)	Cohort	24	4	NASH	CRP	12
Hossain (2016)	(35)	Cross-sectional	63	77	NAFLD	CRP	9
Hui (2004)	(36)	Cross-sectional	48	53	NASH	TNF-α	9
Khoury (2019)	(37)	Cohort	56	35	NASH/Hepatic fibrosis	CRP	12
Klisic (2018)	(38)	Cross-sectional	122	17	NAFLD	CRP	9
Kogiso (2009)	(39)	Cross-sectional	15	60	NAFLD	CRP	8
Koh (2009)	(40)	Cross-sectional	61	42	NAFLD	CRP	9
Koo (2020)	(41)	Cohort	111	119	NASH	CRP	12
Koot (2013)	(42)	Cross-sectional	119	NR	Hepatic steatosis	IL-6/TNF-α	9
Kosmalski (2013)	(43)	Cross-sectional	71	29	NAFLD	CRP	8
Kuppan (2012)	(44)	Case-control	100	100	NAFLD	CRP	9
Lee (2017)	(45)	Cohort	1191	2947	NAFLD	CRP	11
Liang (2015)	(46)	Cross-sectional	105	105	NAFLD	CRP	9
Mahamid (2015)	(47)	Case-control	123	NR	NASH	CRP	8
Mikolasevic (2020)	(48)	Cross-sectional	568	111	NAFLD	CRP	9
Musso (2008)	(49)	Cross-sectional	34	16	NAFLD	ICAM-1	9
Nigam (2013)	(50)	Case-control	120	152	NAFLD	CRP	9
Ogawa (2013)	(51)	Case-control	65	48	NASH	CRP	9
Park (2004)	(52)	Cross-sectional	120	240	NAFLD	CRP	9
Perito (2017)	(53)	Cross-sectional	73	162	NASH	IFN-γ/TGF-β/TNF-α/IL-5/IL-1β/IL-2/IL-4/IL-10/IL-6/IL-7/IL-8/MCP-1	9
Price (2017)	(54)	Cross-sectional	80	446	NAFLD	CRP/ICAM-1/IL-18/IL-6/MCP-1	9
Riquelme (2009)	(55)	Cohort	195	637	NAFLD	CRP	12
Seo (2013)	(56)	Cohort	106	257	NAFLD	TNF-α	12
Shin (2011)	(57)	Cross-sectional	120	28	NAFLD	TNF-α/CRP	9
Shoji (2016)	(58)	Cross-sectional	30	20	Liver cirrhosis	IL-34	9
Simon (2018)	(59)	Cohort	668	3208	NAFLD	IL-6/CRP/IL-2	12
Sung (2009)	(60)	Cross-sectional	2108/3008	7035	Hepatic steatosis	CRP	9
Tabuchi (2010)	(61)	Case-control	42	35	NAFLD	TNF-α	9
Wang (2016)	(62)	Cohort	4304	NR	NAFLD	CRP	12
Wu (2015)	(63)	Case-control	111	120	NAFLD	CRP	9
Yeniova (2014)	(64)	Case-control	210	86	NAFLD	CRP	8
Yoneda (2007)	(65)	Cohort	71	29	NASH	CRP	12
Yu (2018)	(66)	Cross-sectional	7592	12797	NAFLD	CRP	9
Zhu (2008)	(67)	Cross-sectional	48	32	NAFLD	CRP/TNF-α/TGF-β	9
El-Derany (2020)	(68)	Cross-sectional	39	40	NAFLD	IL-13	9
Kumar (2020)	(69)	Cross-sectional	100	100	NAFLD	CRP	9
Ma (2020)	(70)	Cross-sectional	105	213	NAFLD	IL-6	9
Dallio (2021)	(71)	Cross-sectional	30	32	NAFLD	CRP	9
Taniguchi (2021)	(72)	Cross-sectional	3214/2547	NR	NAFLD	CRP	9

NR, not reported; No. of cases, number of cases; No. of controls, number of controls.

### Association Between Inflammatory Cytokines and NAFLD

**Figure 2:**
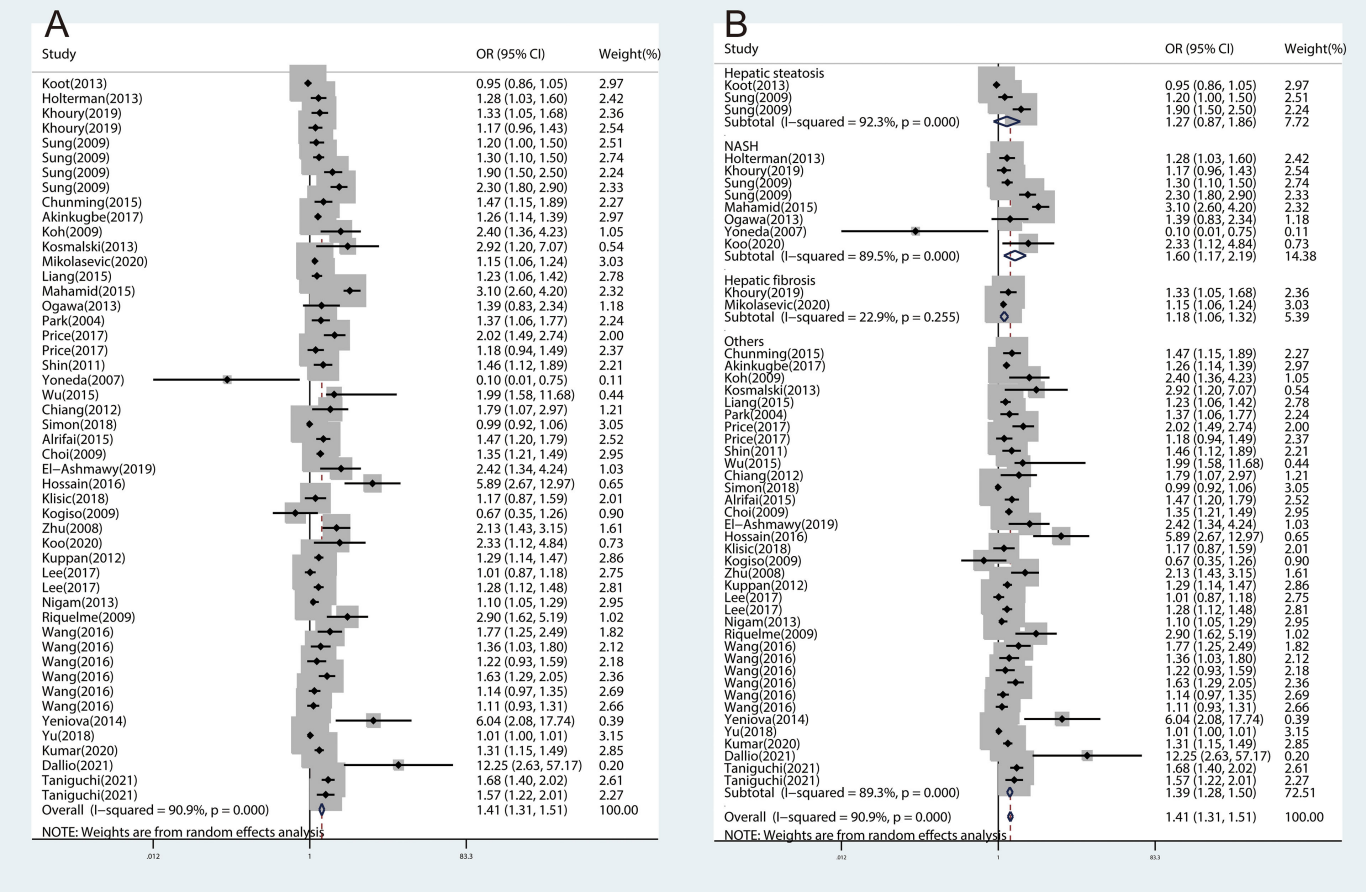
The forest plots of the association between CRP and NAFLD and subtypes of NAFLD. **(A)**, Association between CRP and NAFLD. **(B)**, Association between CRP and subtypes of NAFLD.

**Figure 3:**
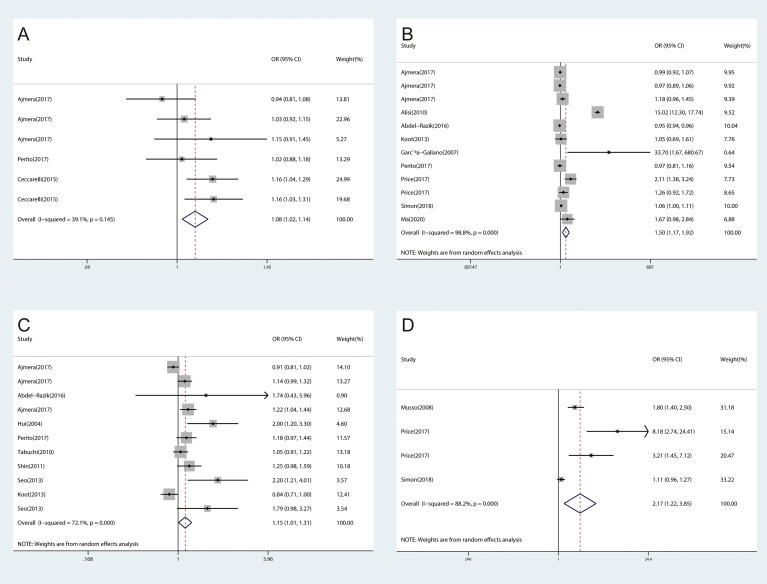
The forest plots of the association between inflammatory cytokines and NAFLD. **(A)**, Association between IL-1β and NAFLD. **(B)**, Association between IL-6 and NAFLD. **(C)**, Association between TNF‐α and NAFLD. **(D)**, Association between ICAM-1 and NAFLD.

The meta-analyses were conducted for 16 inflammatory cytokines, and three inflammatory cytokines, including insulin-like growth factor I (IGF-I), interleukin-13 (IL-13), and interleukin-34 (IL-34), were excluded as they were only reported by one study. We found significant associations between CRP (OR, 1.41; 95% CI, 1.31‐1.51; *P*<0.001) (**Figure 2A**), IL-1β (OR, 1.08; 95% CI, 1.02‐1.14; *P*=0.006) (**Figure 3A**), IL-6 (OR, 1.50; 95% CI, 1.17‐1.92; *P*=0.001) (**Figure 3B**), TNF-α (OR, 1.15; 95% CI, 1.01‐1.31; *P*=0.031) (**Figure 3C**), and ICAM‐1 (OR, 2.17; 95% CI, 1.22‐3.85; *P*=0.008) (**Figure 3D**) and NAFLD. We did not find significant associations between IFN-γ, IGF-II, IL‐2, IL‐4, IL‐5, IL‐7, IL‐8, IL‐10, IL‐12, MCP‐1, and TGF-β and NAFLD (**Supplementary Figures 1–11**).

To further investigate the association between inflammatory cytokines and NAFLD, subgroup analyses were performed by race, mean age, BMI, sample source, diagnostic methods for NAFLD, and the measurements of inflammatory cytokines. Subgroup analyses showed that NAFLD was significantly associated with CRP, TNF-α, and TGF-β in the Asian population. CRP, IL-1β, IL-6, and ICAM-1 were significantly associated with NAFLD in the Caucasian population. When the studies were stratified by mean age, the results showed that the association between IL-1β and NAFLD was statistically significant among studies subjects with age < 18 years. In the 18-60 years group, CRP, TNF-α, TGF-β, and ICAM-1 were significantly associated with NAFLD. In the age ≥ 60 years group, CRP and IL-6 were significantly associated with NAFLD. Besides, CRP, IL-1β, TNF-α and ICAM-1 were associated with NAFLD in the group with BMI < 30. CRP and TGF-β were associated with NAFLD in the group with BMI ≥ 30 (**Table 2** and **Supplementary Table 6**).

**Table 2:** Subgroup analyses of the association between inflammatory cytokines and NAFLD.

Inflammatory cytokines	Subgroup	No. of studies		OR (95%CI)		*P value*		*P* for heterogeneity		*I²* (%)	
**CRP**	**Overall**	49		1.41 (1.31,1.51)		0.000		0.000		90.9	
	**Race**										
	Asian	36		1.44 (1.31,1.59)		0.000		0.000		91.7	
	Caucasian	12		1.29 (1.14,1.47)		0.000		0.000		85.4	
	Others	1		2.42 (1.36,4.31)		0.003		–		–	
	**Mean age**										
	<18	2		1.08 (0.81,1.45)		0.585		0.016		82.9	
	18-60	40		1.45 (1.33,1.58)		0.000		0.000		89.6	
	≥60	5		1.20 (1.03,1.40)		0.021		0.000		81.4	
	Missing	2		1.94 (0.78,4.81)		0.153		0.000		97.6	
	**BMI**										
	<30	33		1.37 (1.25,1.49)		0.000		0.000		89.4	
	≥30	7		1.23 (1.04,1.45)		0.017		0.000		77.9	
	Missing	9		1.73 (1.35,2.23)		0.000		0.000		93.2	
	**Sample**										
	Serum	23		1.34 (1.21,1.47)		0.000		0.000		80.8	
	Plasma	9		1.54 (1.23,1.92)		0.000		0.000		92.9	
	Blood	5		1.75 (1.21,2.55)		0.003		0.000		91.2	
	Missing	12		1.35 (1.17,1.55)		0.000		0.000		82.9	
	**Diagnose**										
	Liver biopsy	11		1.56 (1.19,2.05)		0.001		0.000		84.0	
	US	34		1.39 (1.28,1.50)		0.000		0.000		90.9	
	CT	4		1.34 (0.99,1.80)		0.056		0.000		90.5	
	**Measurement**										
	ELISA	16		1.42 (1.24,1.63)		0.000		0.000		80.1	
	Others	33		1.41 (1.29,1.54)		0.000		0.000		92.4	
						
IL-1β	**Overall**	6		1.08 (1.02,1.14)		0.006		0.145		39.1	
	**Race**										
	Asian	0		–		–		–		–	
	Caucasian	6		1.08 (1.02,1.14)		0.006		0.145		39.1	
	Others	0		–		–		–		–	
	**Mean age**										
	<18	2		1.11 (1.02, 1.21)		0.020		0.171		46.7	
	18-60	3		1.01 (0.93, 1.10)		0.751		0.323		11.6	
	≥60	0		–		–		–		–	
	Missing	1		1.16 (1.03, 1.31)		0.014		–		–	
	**BMI**										
	<30	1		1.16 (1.04, 1.29)		0.007		–		–	
	≥30	3		1.01 (0.93, 1.10)		0.751		0.323		11.6	
	Missing	2		1.10 (1.01, 1.21)		0.039		0.176		45.5	
	**Sample**										
	Serum	0		–		–		–		–	
	Plasma	6		1.08 (1.02,1.14)		0.006		0.145		39.1	
	Blood	0		–		–		–		–	
	Missing	0		–		–		–		–	
	**Diagnose**										
	Liver biopsy	6		1.08 (1.02,1.14)		0.006		0.145		39.1	
	US	0		–		–		–		–	
	CT	0		–		–		–		–	
	**Measurement**										
	ELISA	1		1.02 (0.88, 1.18)		–		0.791		–	
	Others	5		1.09 (1.03, 1.15)		0.108		0.005		47.3	
						
IL-6	**Overall**	12		1.50 (1.17,1.92)		0.001		0.000		98.8	
	**Race**										
	Asian	1		1.67 (0.98,2.84)		0.057		–		–	
	Caucasian	10		1.62 (1.07,2.44)		0.022		0.000		98.9	
	Others	1		0.95 (0.94,0.96)		0.000		–		–	
	**Mean age**										
	<18	3		2.49 (0.33,18.98)		0.378		0.000		99.6	
	18-60	8		1.07 (0.97,1.18)		0.179		0.000		77.8	
	≥60	1		1.06 (1.01,1.12)		0.029		–		–	
	Missing	0		–		–		–		–	
	**BMI**										
	<30	5		2.30 (0.59,8.90)		0.229		0.000		99.6	
	≥30	5		1.03 (0.95,1.11)		0.476		0.028		63.2	
	Missing	2		0.98 (0.83,1.16)		0.827		0.736		0.0	
	**Sample**										
	Serum	6		2.23 (1.37,3.61)		0.001		0.000		99.4	
	Plasma	5		1.01 (0.93,1.10)		0.754		0.151		40.5	
	Blood	0		–		–		–		–	
	Missing	1		1.05 (0.69,1.60)		0.821		–		–	
	**Diagnose**										
	Liver biopsy	7		1.67 (1.08,2.58)		0.022		0.000		99.3	
	US	2		1.29 (0.82,2.02)		0.273		0.180		44.5	
	CT	3		1.34 (0.94,1.91)		0.102		0.004		81.6	
	**Measurement**										
	ELISA	8		1.78 (1.20,2.65)		0.004		0.000		99.2	
	Others	4		1.03 (0.93,1.14)		0.569		0.085		54.7	
						
TNF-α	**Overall**	11		1.15 (1.01,1.31)		0.031		0.000		72.1	
	**Race**									
	Asian	4		1.33 (1.01,1.74)		0.040		0.037		64.7	
	Caucasian	5		1.04 (0.90,1.21)		0.580		0.001		77.4	
	Others	2		1.96 (1.23,3.15)		0.005		0.846		0.0	
	**Mean age**									
	<18	2		0.99 (0.71,1.38)		0.962		0.011		84.6	
	18-60	9		1.21 (1.04,1.40)		0.012		0.001		70.4	
	≥60	0		–		–		–		–	
	Missing	0		–		–		–		–	
	**BMI**									
	<30	5		1.33 (1.03,1.71)		0.028		0.064		55.0	
	≥30	4		1.15 (0.94,1.42)		0.174		0.001		82.0	
	Missing	2		0.99 (0.71,1.38)		0.962		0.011		84.6	
	**Sample**									
	Serum	6		1.44 (1.11,1.87)		0.006		0.022		61.9	
	Plasma	4		1.10 (0.94,1.27)		0.233		0.008		74.4	
	Blood	0		–		–		–		–	
	Missing	1		0.84 (0.71,1.00)		0.046		–		–	
	**Diagnose**									
	Liver biopsy	6		1.16 (0.98,1.36)		0.081		0.003		72.4	
	US	5		1.18 (0.93,1.51)		0.179		0.002		76.9	
	CT	0		–		–		–		–	
	**Measurement**									
	ELISA	8		1.25 (1.02,1.52)		0.030		0.001		71.9	
	Others	3		1.07 (0.89,1.29)		0.444		0.005		80.8	

No. of studies, number of studies; OR (95%CI), odds ratios (95% confidence intervals); US, ultrasonography examination; CT, computed tomography.

Additionally, based on the sample source, the results showed that the associations for CRP, IL-6, TNF-α, TGF‐β, and ICAM-1 with NAFLD were statistically significant among studies in serum samples. Studies using plasma samples suggested significant associations for CRP and IL-1β with NAFLD. Five studies using blood samples showed that CRP was significantly associated with NAFLD. In the subgroup analyses of diagnostic methods for NAFLD and measuring methods for inflammatory cytokines, CRP, IL-1β, and IL-6 were associated with NAFLD among studies with diagnosis by liver biopsy, and CRP, IL-6, and TNF-α were associated with NAFLD among studies with measurement by ELISA (**Table 2** and **Supplementary Table 6**).

Significant heterogeneity was observed in studies investigating the inflammatory cytokines mentioned above (CRP, IL‐6, IL‐8, TNF‐α, TGF‐β, MCP‐1, IGF-II, and ICAM-1), with *I^2^* above 50%, mainly attributed to patient BMI, sample source, and diagnostic methods for NAFLD. In the subgroup analysis of IL‐6, heterogeneity was reduced in the subgroups of the missing BMI group (*I* ^2^ = 0.0%, *P* = 0.736), studies that examined the plasma samples (*I^2^* = 40.5%, *P* = 0.151) and diagnosing NAFLD by ultrasonography examination (*I^2^* = 44.5%, *P* = 0.180). With regard to TGF‐β, heterogeneity was reduced in the BMI ≥ 30 group (*I^2^* = 0.0%, *P* = 0.934). Moreover, heterogeneity was reduced in the serum samples (*I* ^2^ = 0.0%, *P* = 0.480) about MCP-1.

### Association Between Inflammatory Cytokines and Subtypes of NAFLD

**Figure 4:**
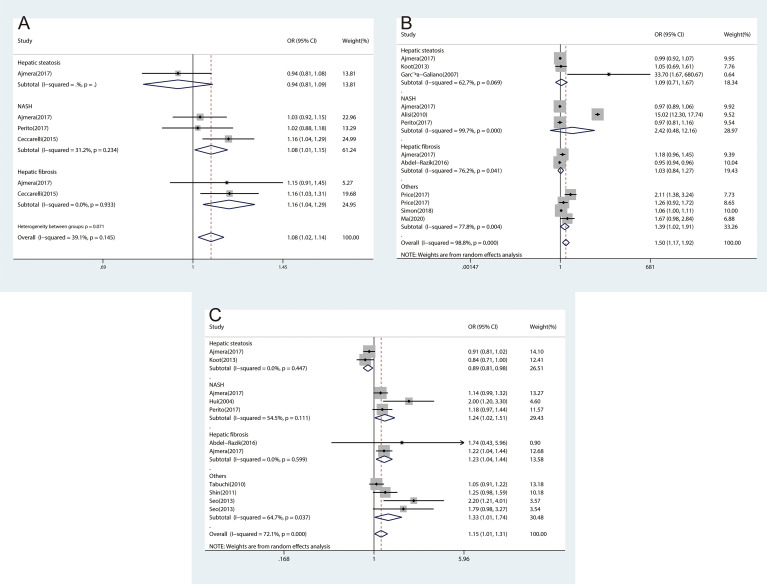
The forest plots of the association between inflammatory cytokines and subtypes of NAFLD. **(A)**, Association between IL-1β and subtypes of NAFLD. **(B)**, Association between IL-6 and subtypes of NAFLD. **(C)**, Association between TNF‐α and subtypes of NAFLD.

Considering NAFLD consists of different clinical subtypes, we performed subgroup analyses by classification of NAFLD as hepatic steatosis, NASH, and hepatic fibrosis. The association between inflammatory cytokines and NAFLD varied among different subtypes of NAFLD. We found significant associations for CRP with NASH (OR, 1.60; 95% CI, 1.17‐2.19; *P*=0.003) and hepatic fibrosis (OR, 1.18; 95% CI, 1.06‐1.32; *P*=0.003), which were consistent with overall effect (**Figure 2B**). The same associations were found in IL-1β (**Figure 4A**) and TNF-α (**Figure 4C**). However, no significant associations were found for IL-6 with NASH and hepatic fibrosis (**Figure 4B**). Besides, we did not find any association between CRP (**Figure 2B**), IL-1β (**Figure 4A**), and IL-6 (**Figure 4B**) and hepatic steatosis with pooled ORs of 1.27 (95% CI, 0.87‐1.86; *P*=0.206), 0.94 (95% CI, 0.81‐1.09; *P*=0.399), and 1.09 (95% CI, 0.71‐1.67; *P*=0.707), respectively.

### Sensitivity Analysis and Publication Bias

We conducted the sensitivity analysis and examined the publication bias of the meta‐analysis on the association between inflammatory cytokines and NAFLD. No individual study markedly affected the pooled OR based on the sensitivity analysis, which indicated that the estimated OR was stable and reliable. Publication bias was assessed using funnel plots, Begg’s tests, and Egger’s tests. There was no significant publication bias detected (**Supplementary Figure 12**).

## Discussion

In this study, 51 studies focusing on the association between inflammatory cytokines and NAFLD were selected and based on which meta‐analyses were conducted. We found significant associations for CRP, IL-1β, IL-6, TNF-α, and ICAM-1 with NAFLD. In contrast, the associations for IFN-γ, IGF-II, IL‐2, IL‐4, IL‐5, IL‐7, IL‐8, IL‐10, IL‐12, MCP‐1, and TGF-β with NAFLD were not significant. For different subtypes of NAFLD, we found significant associations of CRP, IL-1β, and TNF-α with NASH and hepatic fibrosis. And the associations of CRP, IL-1β, and IL-6 with hepatic steatosis were not significant. To the best of our knowledge, this meta‐analysis was the first to comprehensively evaluate the association between inflammatory cytokines and NAFLD.

**Figure 5:**
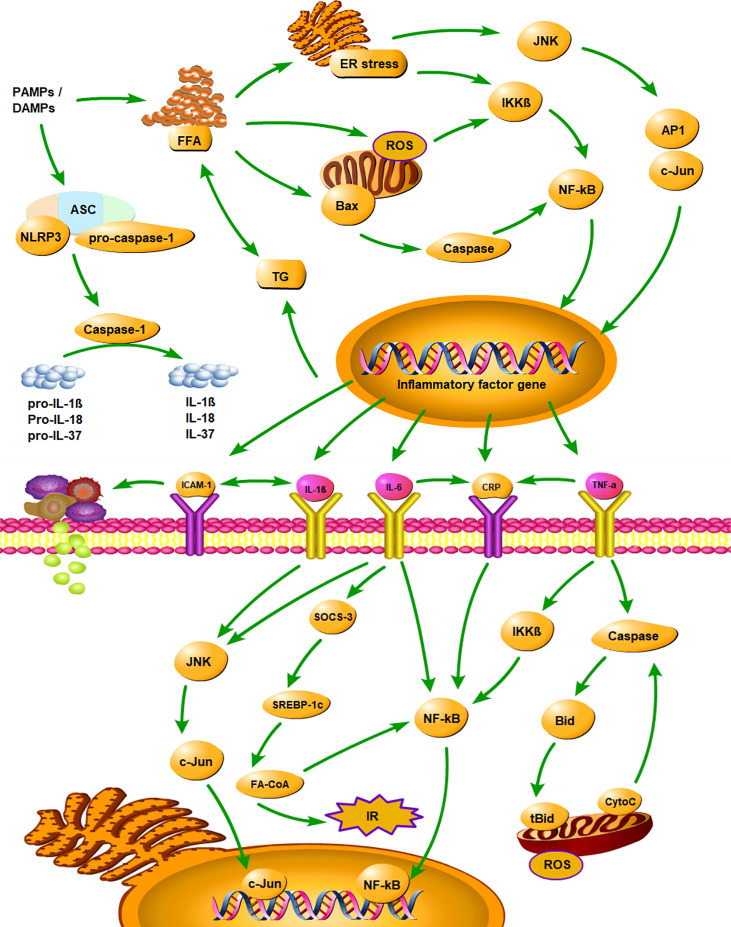
Summarizes the mechanism of inflammatory cytokines and NLRP3 inflammasome in the development of NAFLD. PAMPs, pathogen-associated molecular patterns; DAMPs, danger associated molecular patterns; ASC, apoptosis-associated speck-like protein containing a CARD; CARD, caspase recruitment domain; FFA, free fatty acid; TG, triglyceride; ER stress, endoplasmic reticulum stress; ROS, reactive oxygen Species; IR, insulin resistance; IKKβ, inhibitor kappa B kinase beta; NF-κB, nuclear factor κB; JNK, c-Jun N-terminal kinase; AP1, activator protein 1; SOCS-3, suppressor of cytokine signalling-3; SREBP-1c, sterol regulatory element binding protein-1c; FA-CoA, fatty acyl-CoA; Bid, BH3 interacting-domain death agonist; tBid, truncated BH3 interacting-domain death agonist; CytoC, Cytochrome-C; CRP, C‐reactive protein; IL-1β, interleukin‐1β; IL-6, interleukin‐6; IL-18, interleukin‐18; IL-37, interleukin‐37; TNF-α, tumor necrosis factor‐α; ICAM‐1, intercellular adhesion molecule-1.

It has been shown that the inflammatory cytokines played an important role in the development of NAFLD by activating various inflammatory pathways that interfered with insulin signalling (73). The inhibitor kappa B kinase beta/nuclear factor kappa B (IKK/NF-κB) pathway and the c-Jun N-terminal kinase/activator protein 1 (JNK/AP1) pathway were two well-known pathways (74, 75). Pro-inflammatory cytokines secreted by adipose tissue, such as TNF-α, IL-1β, and IL-6, were involved in the above pathways by activating intracellular kinases, and they also stimulated the production of CRP in the liver (74). Various inflammatory cytokines interacted and inhibited insulin signalling together (73). **Figure 5** illustrates the mechanism of inflammatory cytokines and NLRP3 inflammasome in the development of NAFLD. Among the identified inflammatory cytokines associated with NAFLD, CRP is a classical non-specific acute phase protein produced by the liver (27). Previous study found that CRP can upregulate NF-κB activity, and the activated NF-κB can then join the pathway that interfered with insulin signalling (76). Consistent with the evidence described above, our findings suggested that CRP is closely associated with NAFLD.

TNF-α is a pro-inflammatory cytokine, primarily produced by monocytes and macrophages (77). The release of pro-inflammatory cytokines (TNF-α, IL-1β, IL-6, et al) and reactive oxygen species (ROS) occurred through the pathogen-associated molecular patterns (PAMPs)-toll-like receptors (TLRs)-JNK/NF-κB pathway (78). Hepatic steatosis can lead to an increase in the transcription factor NF-κβ signalling pathway through the upstream activation. Activation of NF-κβ induced production of TNF-α, which helped recruit and activate Kupffer cells to mediate inflammation in NASH (79, 80). Many pieces of evidence indicated a positive association between TNF-α and NAFLD (81). Moreover, studies have shown that TNF-α can be used as a predictor for the development of NAFLD (56, 82). Consistent with these studies (56, 81, 82), the results of the meta-analysis showed that TNF-α can increase the risk of NAFLD.

We also found that IL‐1β, IL‐6, and ICAM-1 were significantly associated with NAFLD, which indicated that these inflammatory cytokines can promote the development of NAFLD. The pro-inflammatory effect of IL-1β may be attributed to its synergistic effect with TLRs signalling, which significantly amplifies inflammation through lipopolysaccharide (LPS) - induced inflammatory mediator (83). The pro-inflammatory effect of IL-6 may be through the suppressor of cytokine signalling-3 (SOCS-3) - sterol regulatory element binding protein-1c (SREBP-1c)- fatty acyl-CoA (FA-CoA) pathway that inhibited insulin signalling and acted to regulate the acute stage response and chronic inflammation (84, 85). Previous research found that macrophages secreted IL-1β and IL-6, which promoted hepatocyte injury and liver fibrosis (18). It has been shown that ICAM-1 was expressed in adipose tissue in addition to lipid-containing hepatocytes and may be a marker of endothelial cell activation (54). In summary, CRP, IL-1β, IL-6, TNF-α, and ICAM-1 may be reliable biomarkers to describe the risk of NAFLD.

Our results showed that there were no significant associations between IFN-γ, IGF-II, IL‐2, IL‐4, IL‐5, IL‐7, IL‐8, IL‐10, IL‐12, MCP‐1, and TGF-β and NAFLD. The main reason for this result may be the limited number of included studies. Besides, the negative findings were strongly linked to multiple characteristics of these inflammatory cytokines involved in the progression of NAFLD. Notably, although the non‐significant associations were observed in the overall population, opposite associations were observed in subgroup analyses. These results may suggest that the true association between inflammatory cytokines and NAFLD was masked or diluted when analyzing the overall population. Therefore, further studies are necessary to establish the true association. Remarkably, the meta-analysis showed that the association between IL-10 and NAFLD reached borderline significance. Due to the limited number of included studies and previous findings, we still considered IL-10 to be an important anti-inflammatory cytokine. IL-10 may have a role by affecting the myeloid-differentiation factor 88 (MyD88)-TLRs-NF-κB signalling pathway (86). Previous research found that IL-10 was a negative regulator in inflammatory response, and it can inhibit the secretion of various inflammatory cytokines by T cells, including IL-2, IL-6, TNF-α, et al (87). Thus, we suggested that this anti-inflammatory cytokine has great potential to help the assessment of NAFLD.

Moreover, recent studies suggested that inflammasome is an intracellular multi-protein complex that is thought to be a trigger for inflammatory cytokines in NAFLD (**Supplementary Table 7**) (88). Although there are different types of inflammasomes, most studies have focused on NLRP3 inflammasomes in NAFLD. It is reported that the activation of NLRP3 inflammasome led to the maturation of caspase-1. On the one hand, caspase-1 may cleave inactive pro-IL-1β, pro-IL-18, and pro-IL-37 to mature IL-1β, IL-18, and IL-37 (17). Increased IL-1β may promote fibrosis directly and induce activation of NF-κB, which may regulate the synthesis of pro-IL-1β, MCP-1, and other inflammatory mediators, leading to a vicious cycle of pro-inflammatory signalling. On the other hand, caspase-1 may generate an N-terminal cleavage product (GSDMD-NT) by cleaving gasdermin-D (GSDMD) specifically, triggering pyroptosis and the release of inflammatory cytokines (89, 90). Therefore, NLRP3 inflammasome is closely associated with the function of inflammatory cytokines in NAFLD and may be a key point in the progression of hepatic steatosis to NASH and hepatic fibrosis.

This study has some potential limitations that should be considered. First, some inflammatory cytokines were only reported by fewer than three studies, thus more studies are needed in the future to confirm this result in different populations. Second, some of the included studies used ultrasonography examination to diagnose NAFLD, which may increase the risk of false positives compared to a diagnosis with the gold standard, i.e. liver biopsy. Finally, there were different cut-off values for inflammatory cytokines in the included studies, which may have increased heterogeneity among studies.

## Conclusion

This review and meta-analysis indicated that increased CRP, IL-1β, IL-6, TNF-α, and ICAM-1 concentrations were significantly associated with increased risks of NAFLD. Furthermore, the results of subgroup analyses showed the characteristics of different inflammatory cytokines involved in the NAFLD progression progress. These inflammatory mediators may serve as biomarkers for the prediction of NAFLD. These findings may provide new insights into the aetiology of NAFLD as well as early diagnosis and intervention.

## Data Availability Statement

The original contributions presented in the study are included in the article/**Supplementary Material**. Further inquiries can be directed to the corresponding author.

## Author Contributions

DY, LuJ, PX and LM designed the study. DY and PX collected the data, and wrote the manuscript with support from other authors. XX and LiJ contributed to the statistical analysis and preparing the tables. LM, LuJ, DY, PX and BPL reviewed and revised the manuscript. All authors approved the final version of the article, including the authorship list.

## Funding

This work was supported by the National Natural Science Foundation of China (81872641) and the Natural Science Foundation of Hunan Province (2021JJ30901).

## Conflict of Interest

The authors declare that the research was conducted in the absence of any commercial or financial relationships that could be construed as a potential conflict of interest.

## Publisher’s Note

All claims expressed in this article are solely those of the authors and do not necessarily represent those of their affiliated organizations, or those of the publisher, the editors and the reviewers. Any product that may be evaluated in this article, or claim that may be made by its manufacturer, is not guaranteed or endorsed by the publisher.
