# Supplementary material for: Association of Inflammatory Cytokines With Non-Alcoholic Fatty Liver Disease
Source: Front Immunol. 2022 May 6;13:880298. doi: 10.3389/fimmu.2022.880298 (PMC9122097; doi:10.3389/fimmu.2022.880298)
Supplement: Supplementary file 1 [file DataSheet_1.docx]

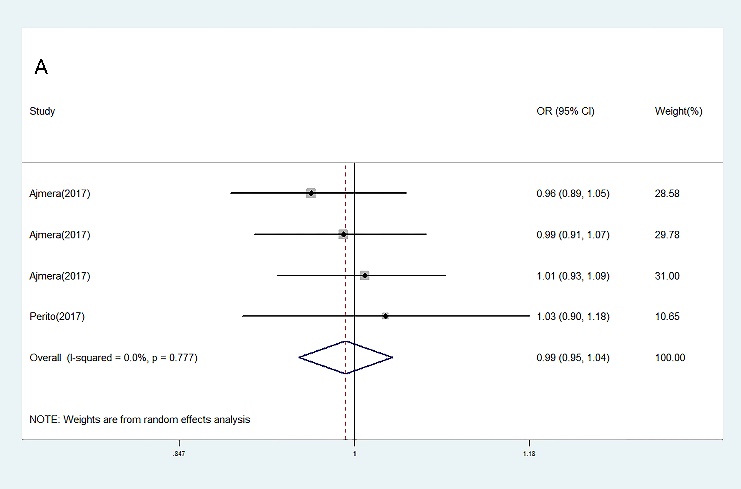

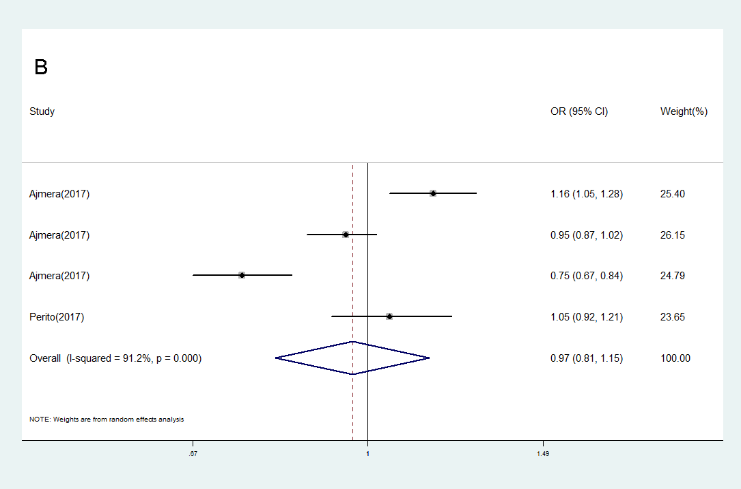


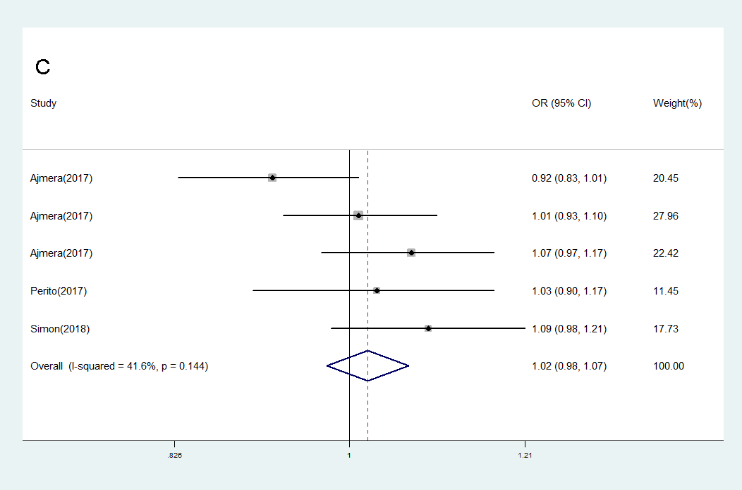

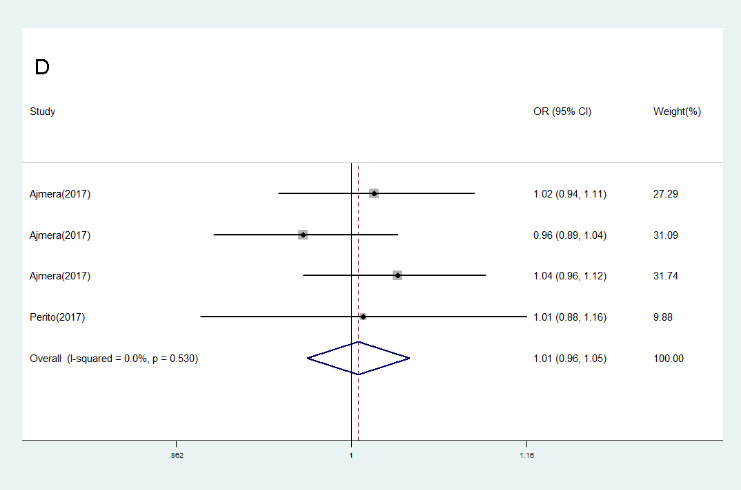

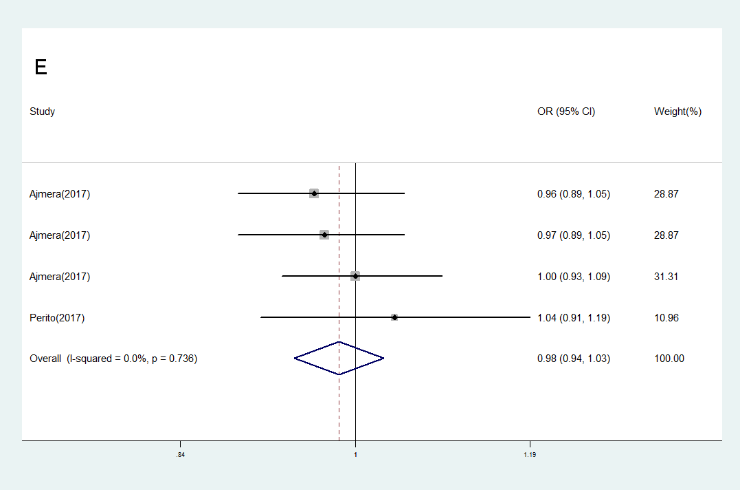

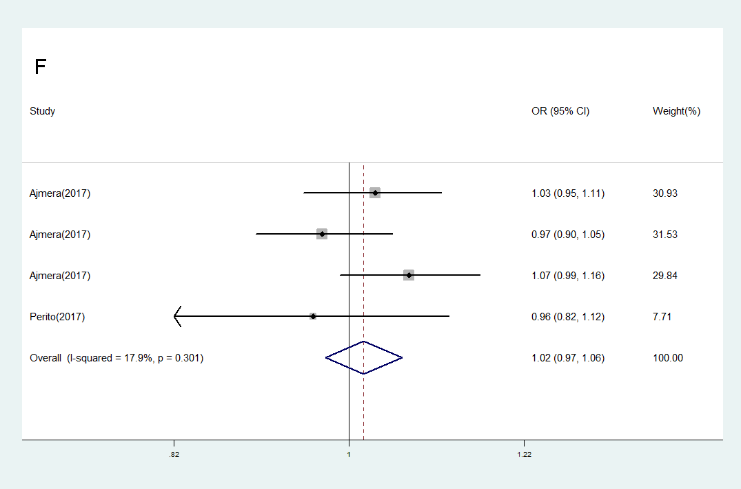

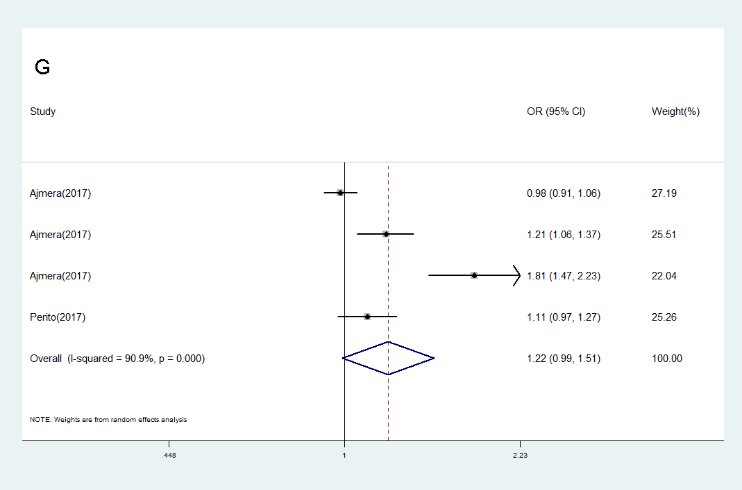

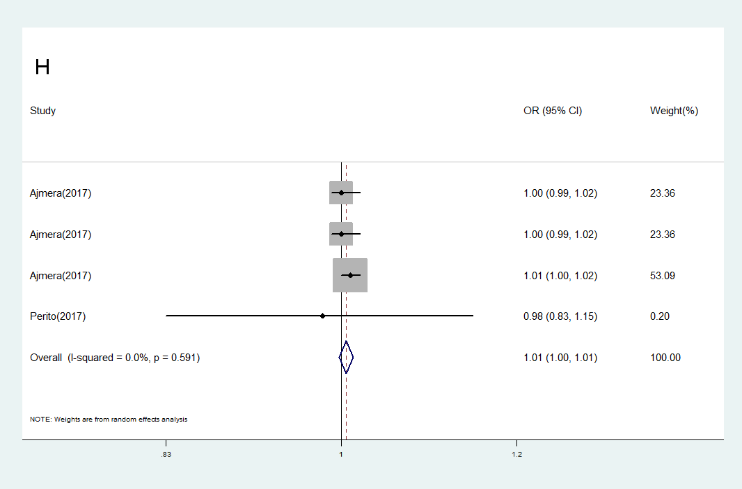

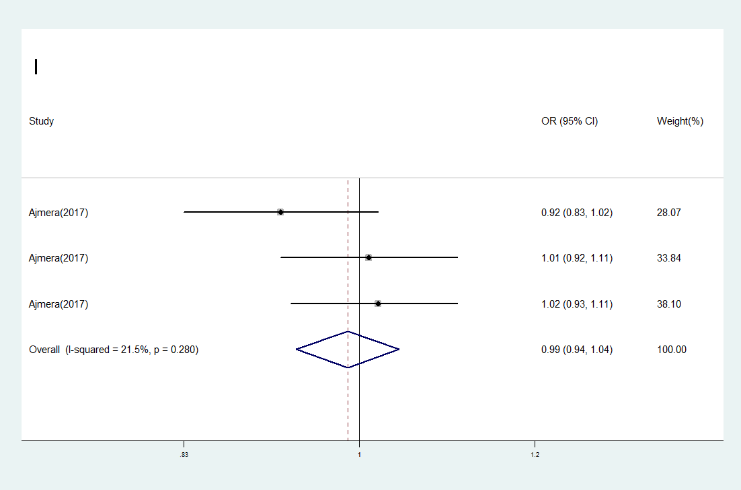

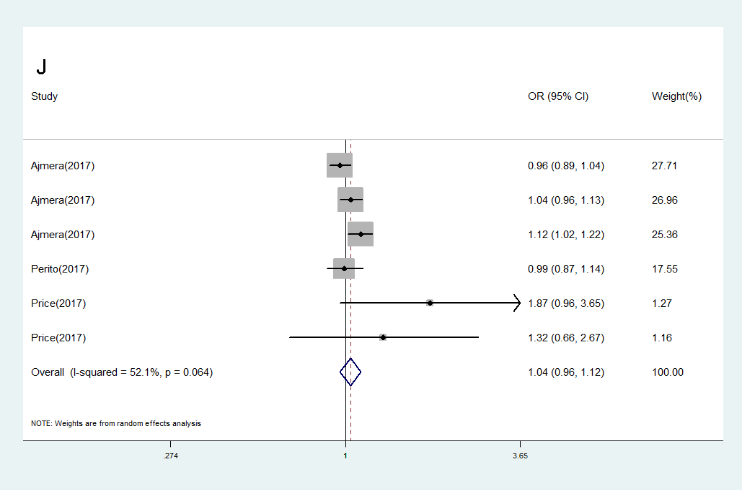

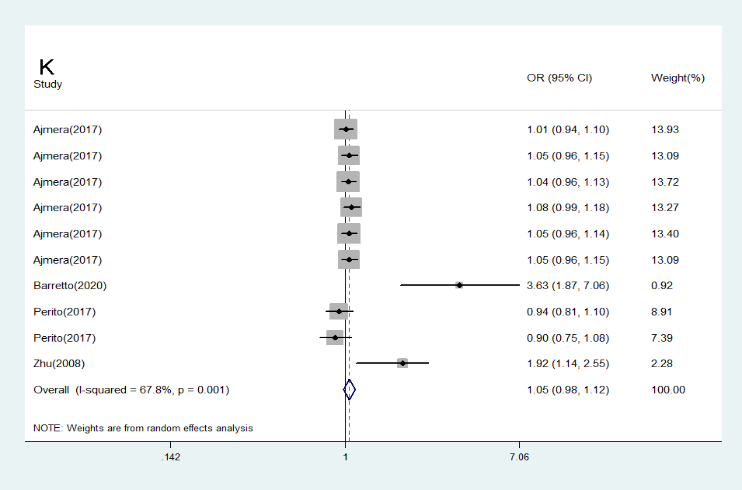


**Supplementary Figure 1-11****.** The forest plots of the association between IFN-γ, IGF-II, IL-2, IL-4, IL-5, IL-7, IL-8, IL-10, IL-12, MCP-1, and TGF-β and NAFLD.


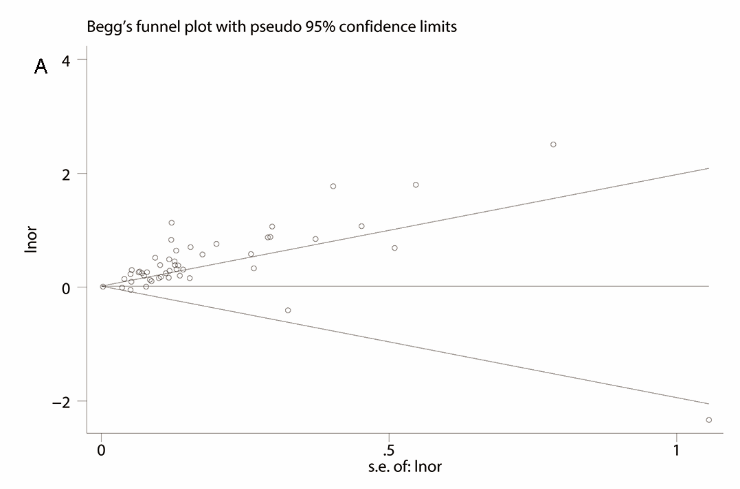

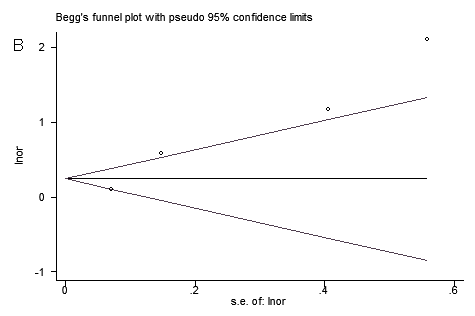

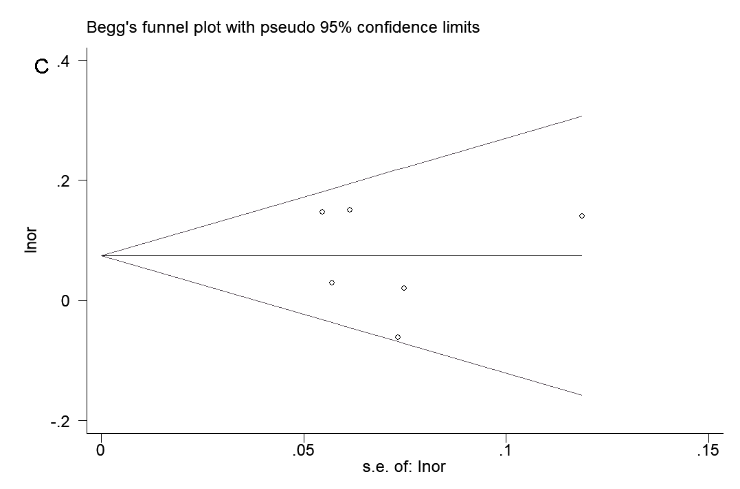

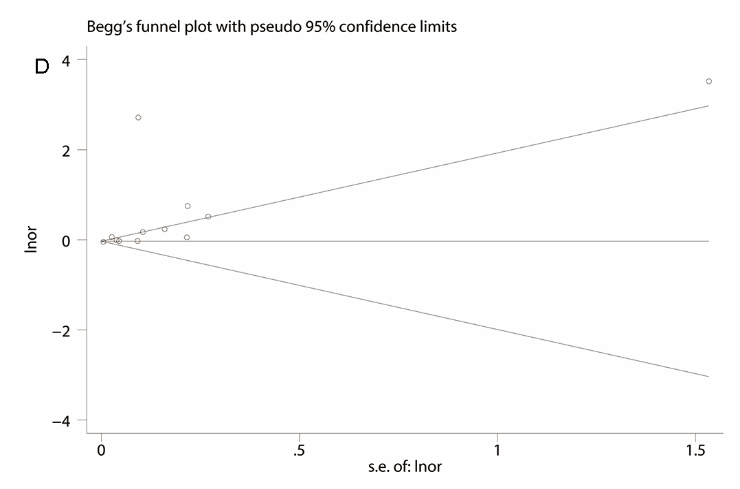

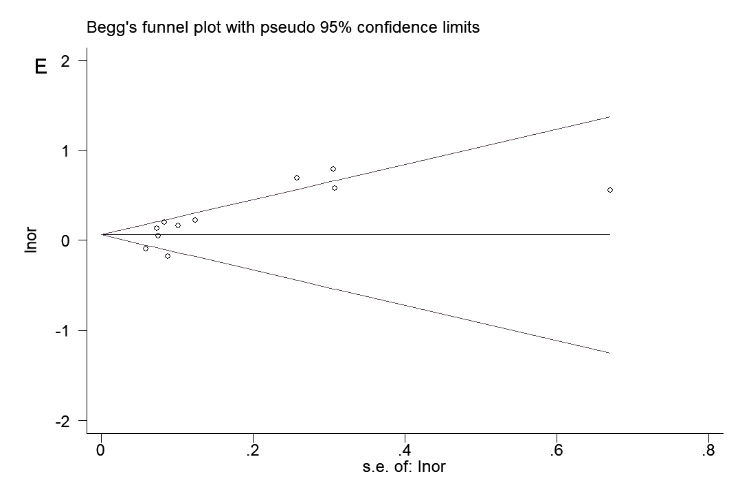


**Supplementary Figure 12a.** The Begg’s tests of the association between CRP, ICAM-1, IL-1β, IL-6, and TNF-α and NAFLD.


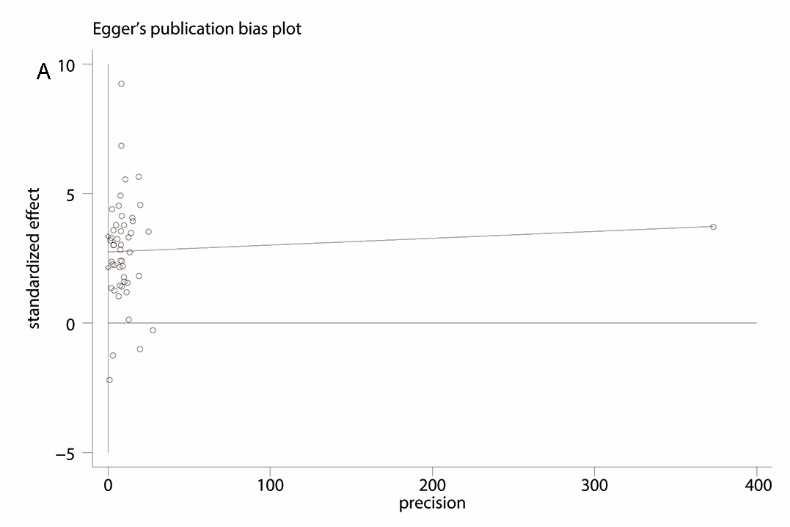

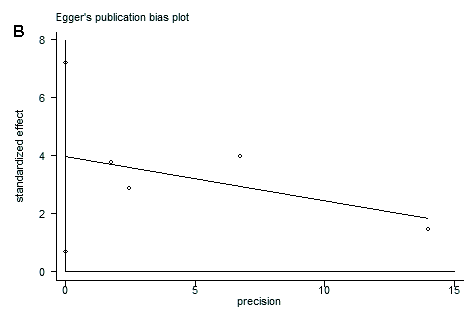

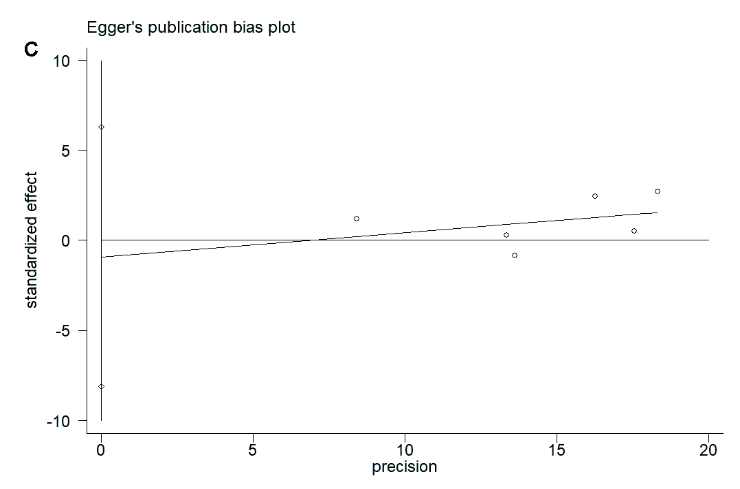

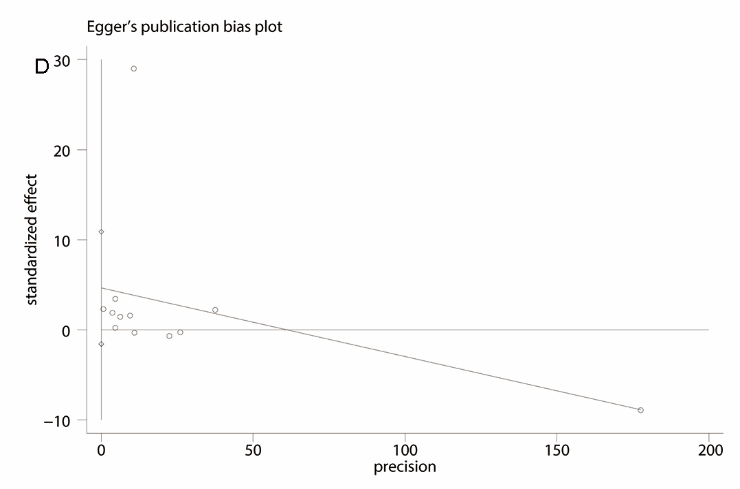

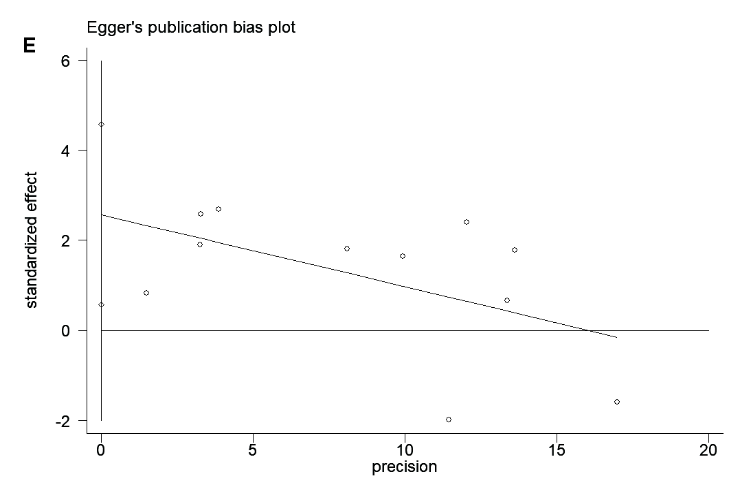


**Supplementary Figure 12b.** The Egger’s tests of the association between CRP, ICAM-1, IL-1β, IL-6, and TNF-α and NAFLD.


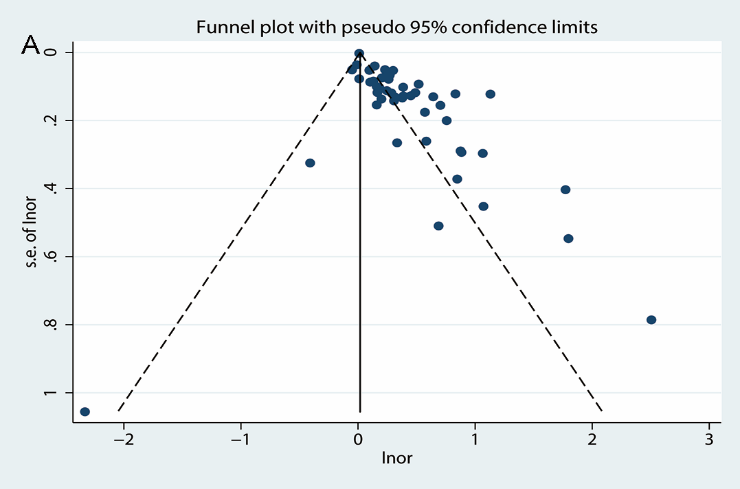

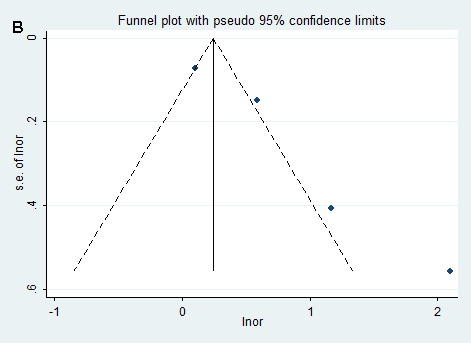

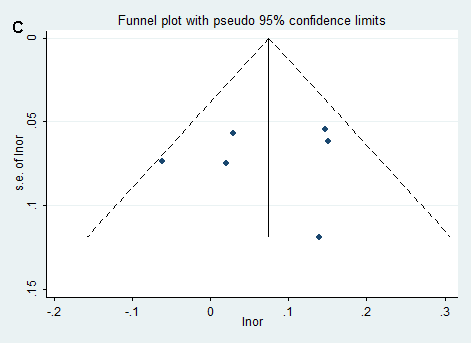

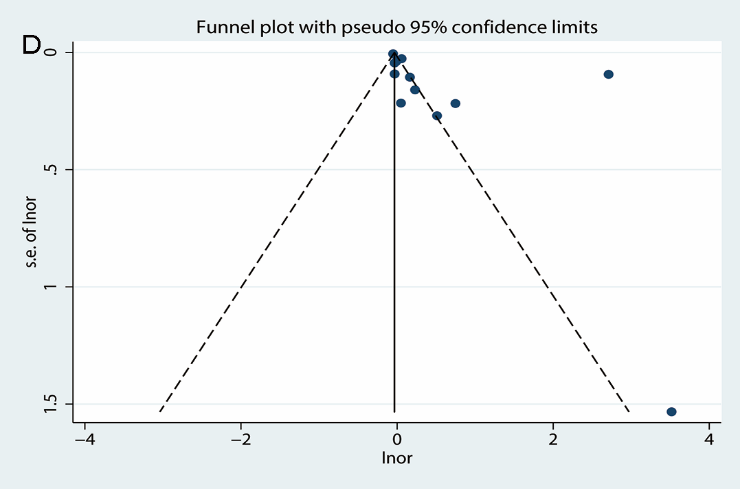

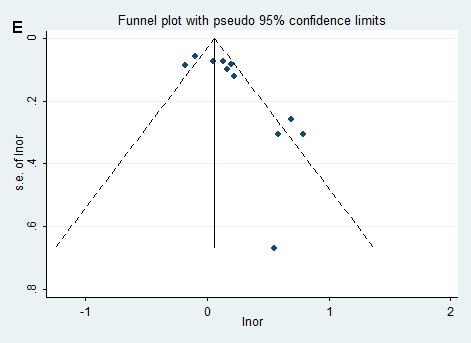


**Supplementary Figure 12c.** The funnel plots of the association between CRP, ICAM-1, IL-1β, IL-6, and TNF-α and NAFLD.


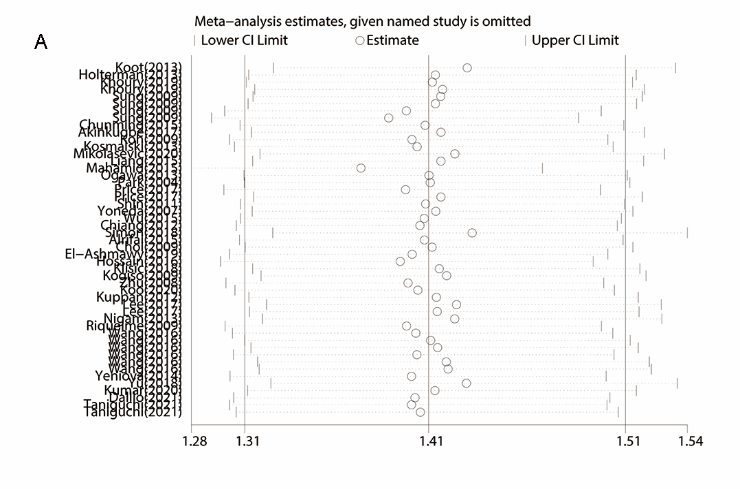

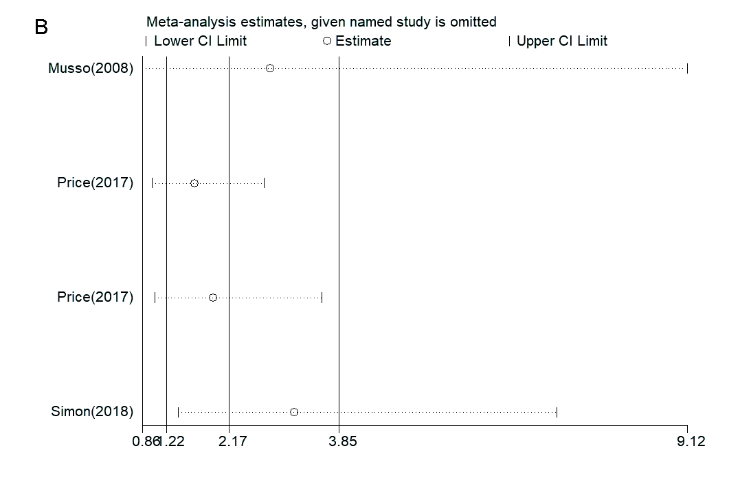

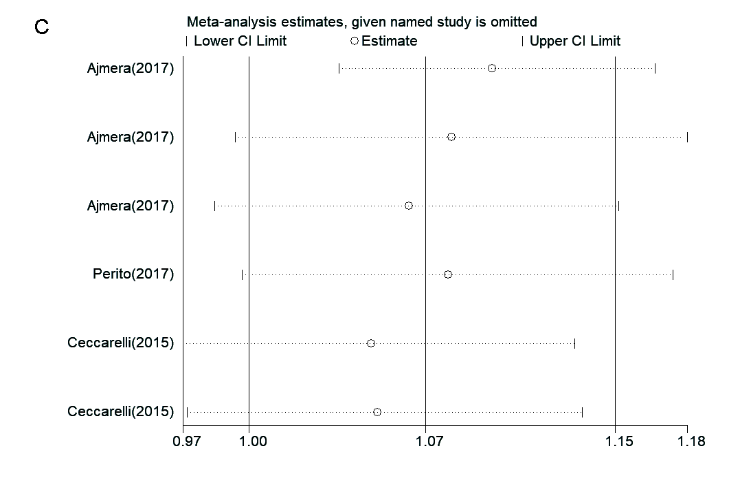

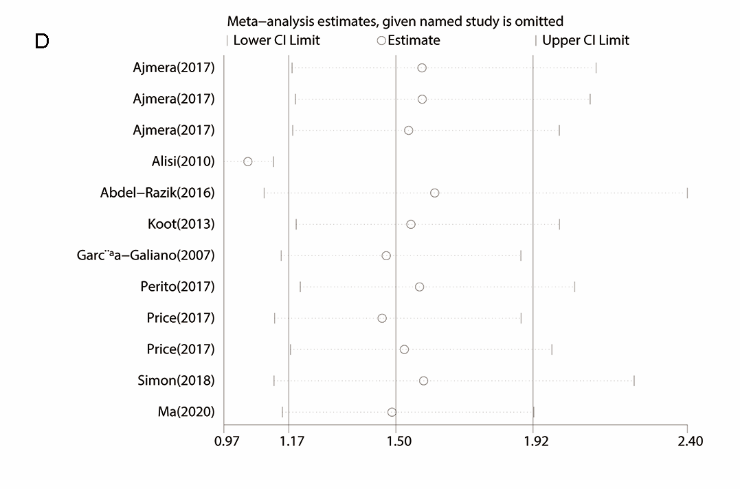

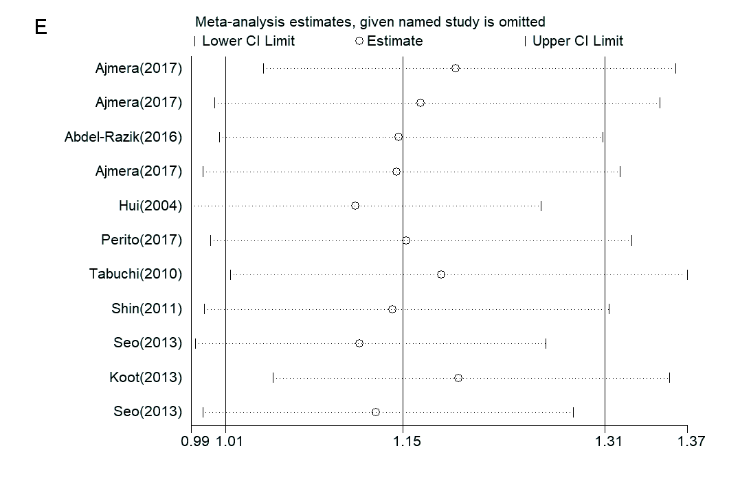


**Supplementary Figure 12d.** The sensitivity analysis of the association between CRP, ICAM-1, IL-1β, IL-6, and TNF-α and NAFLD.

**Supplementary Table 1.** Keywords for the search strategy.

| Trait | Keywords |
| --- | --- |
| Non-alcoholic fatty liver disease | Non-alcoholic fatty liver disease  Non-alcoholic steatohepatitis  Non-alcoholic fatty liver  NAFLD  NASH  NAFL |
| Inflammatory cytokines | Interleukin  Cytokine  Interferon  Lymphocyte  Macrophage  Microglia  Tumor necrosis factor-alpha  C-reactive protein  Transforming growth factor  IFN  IL  CRP  TGF  TNF  Inflammatory factor  Pro-inflammatory cytokine  Chemokine  Inflammatory cytokine |
| Effect estimates | HR or RR or OR |

**Supplementary Table 2.** Quality assessment.

| **First author (year)** | **Study quality assessment criteria** | | | | | | | | | | | | | | **Total score** |
| --- | --- | --- | --- | --- | --- | --- | --- | --- | --- | --- | --- | --- | --- | --- | --- |
|  | **1** | **2** | **3** | **4** | **5** | **6** | **7** | **8** | **9** | **10** | **11** | **12** | **13** | **14** |  |
| Abdel-Razik(2016) | 1 | 1 | 1 | 1 | 0 | 0 | 0 | 1 | 1 | 0 | 1 | 1 | 0 | 1 | 9 |
| Ajmera(2017) | 1 | 1 | 1 | 1 | 0 | 0 | 0 | 1 | 1 | 0 | 1 | 1 | 0 | 1 | 9 |
| Akinkugbe(2017) | 1 | 1 | 1 | 1 | 0 | 0 | 0 | 1 | 1 | 0 | 1 | 0 | 0 | 1 | 8 |
| Alrifai(2015) | 1 | 1 | 1 | 1 | 0 | 0 | 0 | 1 | 1 | 0 | 1 | 1 | 0 | 1 | 9 |
| Alisi(2010) | 1 | 1 | 1 | 1 | 0 | 0 | 0 | 1 | 1 | 0 | 1 | 1 | 0 | 1 | 9 |
| Barretto(2020) | 1 | 1 | 1 | 1 | 0 | 0 | 0 | 1 | 1 | 0 | 1 | 1 | 0 | 1 | 9 |
| Ceccarelli(2015) | 1 | 1 | 1 | 1 | 0 | 1 | 1 | 1 | 1 | 0 | 1 | 1 | 1 | 1 | 12 |
| Chiang(2012) | 1 | 1 | 1 | 1 | 0 | 0 | 0 | 1 | 1 | 0 | 1 | 1 | 0 | 1 | 9 |
| Choi(2009) | 1 | 1 | 1 | 1 | 0 | 0 | 0 | 1 | 1 | 0 | 1 | 1 | 0 | 1 | 9 |
| Chunming(2015) | 1 | 1 | 1 | 1 | 0 | 0 | 0 | 1 | 1 | 0 | 1 | 1 | 0 | 1 | 9 |
| El-Ashmawy(2019) | 1 | 1 | 1 | 1 | 0 | 0 | 0 | 1 | 1 | 0 | 1 | 1 | 0 | 1 | 9 |
| García-Galiano(2007) | 1 | 1 | 1 | 1 | 0 | 0 | 0 | 1 | 1 | 0 | 1 | 1 | 0 | 0 | 8 |
| Holterman(2013) | 1 | 1 | 1 | 1 | 0 | 1 | 1 | 1 | 1 | 0 | 1 | 1 | 1 | 1 | 12 |
| Hossain(2016) | 1 | 1 | 1 | 1 | 0 | 0 | 0 | 1 | 1 | 0 | 1 | 1 | 0 | 1 | 9 |
| Hui(2004) | 1 | 1 | 1 | 1 | 0 | 0 | 0 | 1 | 1 | 0 | 1 | 1 | 0 | 1 | 9 |
| Khoury(2019) | 1 | 1 | 1 | 1 | 0 | 1 | 1 | 1 | 1 | 0 | 1 | 1 | 1 | 1 | 12 |
| Klisic(2018) | 1 | 1 | 1 | 1 | 0 | 0 | 0 | 1 | 1 | 0 | 1 | 1 | 0 | 1 | 9 |
| Kogiso(2009) | 1 | 1 | 0 | 1 | 0 | 0 | 0 | 1 | 1 | 0 | 1 | 1 | 0 | 1 | 8 |
| Koh(2009) | 1 | 1 | 1 | 1 | 0 | 0 | 0 | 1 | 1 | 0 | 1 | 1 | 0 | 1 | 9 |
| Koo(2020) | 1 | 1 | 1 | 1 | 0 | 1 | 1 | 1 | 1 | 0 | 1 | 1 | 1 | 1 | 12 |
| Koot(2013) | 1 | 1 | 1 | 1 | 0 | 0 | 0 | 1 | 1 | 0 | 1 | 1 | 0 | 1 | 9 |
| Kosmalski(2013) | 1 | 1 | 1 | 1 | 0 | 0 | 0 | 1 | 1 | 0 | 1 | 1 | 0 | 0 | 8 |
| Kuppan(2012) | 1 | 1 | 1 | 1 | 0 | 0 | 0 | 1 | 1 | 0 | 1 | 1 | 0 | 1 | 9 |
| Lee(2017) | 1 | 1 | 0 | 1 | 0 | 1 | 1 | 1 | 1 | 0 | 1 | 1 | 1 | 1 | 11 |
| Liang(2015) | 1 | 1 | 1 | 1 | 0 | 0 | 0 | 1 | 1 | 0 | 1 | 1 | 0 | 1 | 9 |
| Mahamid(2015) | 1 | 1 | 1 | 1 | 0 | 0 | 0 | 1 | 1 | 0 | 1 | 1 | 0 | 0 | 8 |
| Mikolasevic(2020) | 1 | 1 | 1 | 1 | 0 | 0 | 0 | 1 | 1 | 0 | 1 | 1 | 0 | 1 | 9 |
| Musso(2008) | 1 | 1 | 1 | 1 | 0 | 0 | 0 | 1 | 1 | 0 | 1 | 1 | 0 | 1 | 9 |
| Nigam(2013) | 1 | 1 | 1 | 1 | 0 | 0 | 0 | 1 | 1 | 0 | 1 | 1 | 0 | 1 | 9 |
| Ogawa(2013) | 1 | 1 | 1 | 1 | 0 | 0 | 0 | 1 | 1 | 0 | 1 | 1 | 0 | 1 | 9 |
| Park(2004) | 1 | 1 | 1 | 1 | 0 | 0 | 0 | 1 | 1 | 0 | 1 | 1 | 0 | 1 | 9 |
| Perito(2017) | 1 | 1 | 1 | 1 | 0 | 0 | 0 | 1 | 1 | 0 | 1 | 1 | 0 | 1 | 9 |
| Price(2017) | 1 | 1 | 1 | 1 | 0 | 0 | 0 | 1 | 1 | 0 | 1 | 1 | 0 | 1 | 9 |
| Riquelme(2009) | 1 | 1 | 1 | 1 | 0 | 1 | 1 | 1 | 1 | 0 | 1 | 1 | 1 | 1 | 12 |
| Seo(2013) | 1 | 1 | 1 | 1 | 0 | 1 | 1 | 1 | 1 | 0 | 1 | 1 | 1 | 1 | 12 |
| Shin(2011) | 1 | 1 | 1 | 1 | 0 | 0 | 0 | 1 | 1 | 0 | 1 | 1 | 0 | 1 | 9 |
| Shoji(2016) | 1 | 1 | 1 | 1 | 0 | 0 | 0 | 1 | 1 | 0 | 1 | 1 | 0 | 1 | 9 |
| Simon(2018) | 1 | 1 | 1 | 1 | 0 | 1 | 1 | 1 | 1 | 0 | 1 | 1 | 1 | 1 | 12 |
| Sung(2009) | 1 | 1 | 1 | 1 | 0 | 0 | 0 | 1 | 1 | 0 | 1 | 1 | 0 | 1 | 9 |
| Tabuchi(2010) | 1 | 1 | 1 | 1 | 0 | 0 | 0 | 1 | 1 | 0 | 1 | 1 | 0 | 1 | 9 |
| Wang(2016) | 1 | 1 | 1 | 1 | 0 | 1 | 1 | 1 | 1 | 0 | 1 | 1 | 1 | 1 | 12 |
| Wu(2015) | 1 | 1 | 1 | 1 | 0 | 0 | 0 | 1 | 1 | 0 | 1 | 1 | 0 | 1 | 9 |
| Yeniova(2014) | 1 | 1 | 1 | 1 | 0 | 0 | 0 | 1 | 1 | 0 | 1 | 1 | 0 | 0 | 8 |
| Yoneda(2007) | 1 | 1 | 1 | 1 | 0 | 1 | 1 | 1 | 1 | 0 | 1 | 1 | 1 | 1 | 12 |
| Yu(2018) | 1 | 1 | 1 | 1 | 0 | 0 | 0 | 1 | 1 | 0 | 1 | 1 | 0 | 1 | 9 |
| Zhu(2008) | 1 | 1 | 1 | 1 | 0 | 0 | 0 | 1 | 1 | 0 | 1 | 1 | 0 | 1 | 9 |
| El-Derany(2020) | 1 | 1 | 1 | 1 | 0 | 0 | 0 | 1 | 1 | 0 | 1 | 1 | 0 | 1 | 9 |
| Kumar(2020) | 1 | 1 | 1 | 1 | 0 | 0 | 0 | 1 | 1 | 0 | 1 | 1 | 0 | 1 | 9 |
| Ma(2020) | 1 | 1 | 1 | 1 | 0 | 0 | 0 | 1 | 1 | 0 | 1 | 1 | 0 | 1 | 9 |
| Dallio(2021) | 1 | 1 | 1 | 1 | 0 | 0 | 0 | 1 | 1 | 0 | 1 | 1 | 0 | 1 | 9 |
| Taniguchi(2021) | 1 | 1 | 1 | 1 | 0 | 0 | 0 | 1 | 1 | 0 | 1 | 1 | 0 | 1 | 9 |

**Supplementary Table 3**. Characteristics of included studies.

| **Study (Year)** | **Reference** | **Case type** | **Control type** | **Risk factor** | **Adjustment factors** | **Complications** | **Associated diseases** |
| --- | --- | --- | --- | --- | --- | --- | --- |
| Abdel-Razik (2016) | (1) | HB | NR | NR | age, sex, BMI, platelet, ALT, AST, ALP, γ-GT, Albumin, MPV, N/L ratio | NR | NR |
| Ajmera (2017) | (2) | HB | HB | IGFII (per 0.55ng/mL)  IL-8 (per 2.35 pg/mL)  MCP-1(per45.4pg/mL)  TNFα (per3.75 pg/mL) | sex, age, BMI, AST, ALT, HDL-C, HOMA-IR, TG | NR | NR |
| Akinkugbe (2017) | (3) | PB | PB | CRP＜30% | age, sex, WC, smoking, physical activity, alcohol consumption, and diabetes | NR | periodontitis |
| Alisi (2010) | (4) | HB | NR | NR | NR | NR | NR |
| Alrifai (2015) | (5) | PB | PB | CRP≥2mg/L | age, gender, ethnicity, smoking status, LDL cholesterol, use of lipid-lowering medication, highest level of education completed, obesity and components of metabolic syndrome | atherosclerosis | NR |
| Barretto (2020) | (6) | HB | HB | NR | BMI/age Z-score and WC | NR | NR |
| Ceccarelli (2015) | (7) | HB | HB | NR | NR | NR | NR |
| Chiang (2012) | (8) | HB | HB | NR | metabolic syndrome and uric acid levels | NR | NR |
| Choi (2009) | (9) | PB | PB | NR | NR | NR | coronary heart disease |
| Chunming (2015) | (10) | HB | HB | NR | NR | NR | NR |
| El-Ashmawy (2019) | (11) | HB | HB | NR | age and sex | NR | type 2 diabetes |
| García-Galiano (2007) | (12) | HB | NR | IL-6 >4.81pg/mL  IGF-I<130 ng/mL | NR | morbidly obese | NR |
| Holterman (2013) | (13) | HB | NR | NR | NR | severely obese | NR |
| Hossain (2016) | (14) | HB | HB | NR | BMI, TG, HOMA-IR | NR | prediabetes |
| Hui (2004) | (15) | HB | PB | NR | insulin resistance | NR | NR |
| Khoury (2019) | (16) | HB | HB | NR | NR | NR | NR |
| Klisic (2018) | (17) | PB | PB | NR | age, body height, diabetes duration (all continuous variables), gender, smoking habits, therapies (all categorical variables) | NR | type 2 diabetes |
| Kogiso (2009) | (18) | HB | HB | CRP＜3 mg/L | BMI, WC, BW, TG, W/H ratio, Hip circumference, HDL-C, FBG, systolic blood pressure, diastolic blood pressure | NR | NR |
| Koh (2009) | (19) | HB | HB | NR | age and sex | NR | type 2 diabetes |
| Koo (2020) | (20) | HB | HB | hsCRP≥1 mg/L | PNPLA3 genotype, TM6SF2 genotype, Diabetes mellitus, AST, HOMA-IR | NR | diabetes |
| Koot (2013) | (21) | HB | NR | NR | NR | NR | NR |
| Kosmalski (2013) | (22) | HB | NR | CRP≥0.48 mg/dL | NR | NR | type 2 diabetes |
| Kuppan (2012) | (23) | PB | PB | NR | waist circumference, IR, serum TG, and presence of type 2 diabetes | NR | NR |
| Lee (2017) | (24) | PB | PB | 0–0.2 mg/L、  0.3-0.4 mg/L、  0.5-0.9 mg/L | age, smoking, exercise, BMI, TG, SBP, and fasting serum glucose | NR | NR |
| Liang (2015) | (25) | HB | HB | NR | NR | NR | coronary heart disease |
| Mahamid (2015) | (26) | HB | NR | NR | NR | NR | hyperplastic polyps |
| Mikolasevic (2020) | (27) | HB | NR | NR | NR | NR | type 2 diabetes |
| Musso (2008) | (28) | HB | HB | NR | NR | metabolic syndrome | NR |
| Nigam (2013) | (29) | PB | PB | CRP≥1mg/L | BMI, WC, FBG, TC, TG, blood pressure, the metabolic syndrome | NR | NR |
| Ogawa (2013) | (30) | PB | HB | NR | age, gender, BMI, ALT, Scd14 | NR | NR |
| Park (2004) | (31) | HB | PB | CRP (per 1.48 mg/L) | BMI, HDL-C, HOMA-IR | NR | NR |
| Perito (2017) | (32) | HB | NR | NR | NR | NR | NR |
| Price (2017) | (33) | PB | NR | NR | NR | NR | HIV |
| Riquelme (2009) | (34) | HB | HB | CRP＞0.86 mg/L | ALT, BMI | NR | NR |
| Seo (2013) | (35) | PB | PB | TNF-α (2.4-3.8)  TNF-α (3.8-12) | age, BMI, and smoking | NR | NR |
| Shin (2011) | (36) | HB | HB | NR | age and/or gender | NR | type 2 diabetes |
| Shoji (2016) | (37) | HB | PB | NR | NR | NR | NR |
| Simon (2018) | (38) | HB | HB | NR | age, sex, ethnicity, MESA study site, smoking history, alcohol intake (servings/day), BMI, diabetes, SBP, use of anti-hypertensive medications, TC, HDL-C, use of lipid-lowering medications and physical activity | atherosclerosis | NR |
| Sung (2009) | (39) | HB | HB | CRP (>2 mg/L) | age, BMI, smoking, exercise (except 10-year risk score ≥10%) | NR | cardiovascular disease |
| Tabuchi (2010) | (40) | HB | HB | NR | BMI | NR | NR |
| Wang (2016) | (41) | PB | NR | Q1(0-0.1nmol/L)、  Q3(0.5-0.8nmol/L) 、Q4(0.9-25.9nmol/L) | age, BMI, SBP, DBP, HDL-C, LDL-C, TG, TC, creatinine, uric acid, FBG, blood urea nitrogen, hemoglobin, height and weight | NR | NR |
| Wu (2015) | (42) | HB | PB | NR | BMI, TC, HOMA-IR, SOD, CgA | NR | NR |
| Yeniova (2014) | (43) | HB | HB | NR | NR | NR | NR |
| Yoneda (2007) | (44) | HB | HB | NR | age, sex, presence of diabetes,  HOMA-IR, serum TG, HDL-C and LDL-C, BMI, VFA, and SFA | NR | NR |
| Yu (2018) | (45) | HB | HB | NR | sex, age, smoking, BMI, HOMA-IR, γ-GT, TG, FPG | NR | NR |
| Zhu (2008) | (46) | HB | HB | NR | BMI, ALT, TG, TC, FBG, mean blood pressure | NR | NR |
| El-Derany (2020) | (47) | HB | HB | NR | NR | NR | hepatocellular  carcinoma |
| Kumar (2020) | (48) | HB | HB | NR | waist‑hip circumference ratio and HbA1C | NR | NR |
| Ma (2020) | (49) | PB | PB | NR | age, gender, BMI, WC, UA, glucose tolerance status, HOMA-IR,blood pressure, lipids, ALT, AST, and mtDNAcn | NR | NR |
| Dallio (2021) | (50) | HB | HB | NR | NR | NR | NR |
| Taniguchi (2021) | (51) | HB | HB | NR | age, BMI, status of alcohol drinking and smoking, presence of hyperlipidemia, glucose intolerance, and hyper-tension | NR | NR |

Abbreviation: NR, not reported; HB, hospital-based; PB, population-based; BMI, body mass index; ALP, alkaline phosphatase; γ-GT, γ-glutamyl transpeptidase; MPV, mean platelet volume; N/L ratio, neutrophil-lymphocyte ratio; TC, total cholesterol; TG, Triglycerides; LDL-C, low density lipoprotein cholesterol; HDL-C, high-density lipoprotein cholesterol; ALT, alanine aminotransferase; AST, aspartate aminotransferase; MS, metabolic syndrome; IR, insulin resistance; HOMA-IR, homeostasis model assessment of insulin resistance index; BW, body weight; WC, waist circumference; W/H ratio, waist circumference/hip circumference ratio; FBG, fasting blood glucose; SBP, systolic blood pressure; DBP, diastolic blood pressure; SOD, superoxide dismutase; CgA, chromogranin A;VFA, visceral fat area; SFA, subcutaneous fat area.

**Supplementary Table 4**. Clinical indicators of included studies.

| **Study (Year)** | **Reference** | **Material** | | **Race** | **Male gender**  **n (%)** | **BMI** | **Mean age** | **Diagnosis**  **of NAFLD** | **Measurement of inflammatory cytokines** |
| --- | --- | --- | --- | --- | --- | --- | --- | --- | --- |
| Abdel-Razik (2016) | (1) | Serum | African | | 24(20) | 29.9±2.8 | 49(41-57) | Liver biopsy | ELISA |
| Ajmera (2017) | (2) | Plasma | North American | | 126(33.5) | 34.5±6.1 | 47.7 | Liver biopsy | Luminex Multiplex |
| Akinkugbe (2017) | (3) | Serum | European | | 376(15) | NR | 56 | US | TIIA |
| Alisi (2010) | (4) | Serum | European | | 27(67.5) | 94.6±5.6 | 11.9±2.8 | Liver biopsy | ELISA |
| Alrifai (2015) | (5) | Serum | North American | | 305(45.5) | NR | 61.2±9.6 | CT | TIIA |
| Barretto (2020) | (6) | Plasma | South American | | 20(55.7) | NR | 14(12-16) | US | ELISA |
| Ceccarelli (2015) | (7) | Plasma | European | | 27(67.5) | 29.4±10.6 | 12±2.6 | Liver biopsy | Cytokine Antibody Array |
| Chiang (2012) | (8) | Plasma | Asian | | 16(47) | 26.7±5.3 | 71±15 | US | TIIA |
| Choi (2009) | (9) | Serum | Asian | | 4246(73.6) | 25.4±2.7 | 51.7±10.7 | US | NR |
| Chunming (2015) | (10) | Blood | Asian | | 17(60.7） | 27.97±1.55 | 47.17±13.17 | US | NR |
| El-Ashmawy (2019) | (11) | Serum | African | | 46(49.5) | 27.8±4.1 | 53.4±9.2 | US | ELISA |
| García-Galiano (2007) | (12) | Serum | European | | NR | 51±7.0 | 44±11.0 | Liver biopsy | ELISA |
| Holterman (2013) | (13) | Serum | North American | | 5(21) | 52±10 | 16±1 | Liver biopsy | ELISA |
| Hossain (2016) | (14) | Serum | Asian | | 42(54.5) | 26.68±4.16 | 46.1±9.2 | US | ELISA |
| Hui (2004) | (15) | Serum | Australia | | 48(100) | 30.7±0.7 | 41.2±2.7 | Liver biopsy | ELISA |
| Khoury (2019) | (16) | Serum | Asian | | NR | 28.5±3.1 | 43.8±11.8 | Liver biopsy | NR |
| Klisic (2018) | (17) | Serum | European | | 62(50.8) | 31.1(28.7-34.1) | 63(55-69) | FLI-score | TIIA |
| Kogiso (2009) | (18) | Serum | Asian | | 15(100) | 25.0±2.5 | 55.8±11.6 | US | latex agglutination assay |
| Koh (2009) | (19) | Blood | Asian | | 28(45.9) | NR | 55.2±11.8 | US | ELISA |
| Koo (2020) | (20) | NR | Asian | | NR | 27.9(25.4-31.8) | 53.8±15.1 | Liver biopsy | NR |
| Koot (2013) | (21) | NR | European | | 48(41) | NR | 14.3±2.1 | US | ELISA |
| Kosmalski (2013) | (22) | Serum | European | | NR | 31.1±5.62 | 56.37±13.51 | US | NR |
| Kuppan (2012) | (23) | Plasma | Asian | | NR | 25±3.9 | 43±12.4 | US | TIIA |
| Lee (2017) | (24) | Serum | Asian | | NR | 22.5(2.2) | 36.5(4.7) | US | TIIA |
| Liang (2015) | (25) | Blood | Asian | | NR | 27.193±3.045 | NR | US | NR |
| Mahamid (2015) | (26) | Blood | Asian | | NR | NR | NR | Liver biopsy | NR |
| Mikolasevic (2020) | (27) | NR | European | | 306(53.9) | 31.46±5.03 | 65.19±11.01 | US | NR |
| Musso (2008) | (28) | Serum | European | | 29(85.3) | 25.6±2.1 | 45±10 | US | ELISA |
| Nigam (2013) | (29) | Serum | Asian | | 92(76.7) | 27.9±3.6 | 38.5±8.8 | US | ELISA |
| Ogawa (2013) | (30) | Serum | Asian | | 36(55.4) | 29.1±5.1 | 51.4±12.8 | Liver biopsy | ELISA |
| Park (2004) | (31) | Blood | Asian | | NR | 23.4±0.1 | 42.8±0.88 | US | TIIA |
| Perito (2017) | (32) | Plasma | North American | | NR | NR | 13.5±2.3 | Liver biopsy | ELISA |
| Price (2017) | (33) | Serum | North American | | 80(100) | 28(26-32) | 54(48-59) | CT | TIIA/ELISA |
| Riquelme (2009) | (34) | Serum | South American | | 69(35.4) | NR | 50.2±11.1 | US | NR |
| Seo (2013) | (35) | Serum | Asian | | 79(74.5) | 23.4±2.7 | 40.9(35-45) | US | ELISA |
| Shin (2011) | (36) | Serum | Asian | | NR | 26.8±3.4 | 55.1±8.7 | US | ELISA |
| Shoji (2016) | (37) | Serum | Asian | | 9(30) | 27.9(20.1-38.6) | 66(47-80) | Liver biopsy | NR |
| Simon (2018) | (38) | Serum | North American | | 298(44.6) | 31.2±5.5 | 61.2±9.6 | CT | ELISA |
| Sung (2009) | (39) | Plasma | Asian | | 2108(100) | 25.2(25.1-25.3) | 41.7±8.8 | US | TIIA |
| Tabuchi (2010) | (40) | Serum | Asian | | 42(100) | 26.1±2.7 | 51.6±6.3 | US | ELISA |
| Wang (2016) | (41) | NR | Asian | | 502(100) | 22.23±2.61 | 38.2±11.32 | US | ELISA |
| Wu (2015) | (42) | Plasma | Asian | | 64(57.7） | 30.0±9.1 | 53.7±11.8 | US | ELISA |
| Yeniova (2014) | (43) | Plasma | Asian | | 68(32.4) | 31.88±0.5 | 46.04±0.75 | US | TIIA |
| Yoneda (2007) | (44) | Serum | Asian | | 32(45.1) | 28.4±5.2 | 50.9±13.7 | Liver biopsy | RT-PCR |
| Yu (2018) | (45) | Plasma | Asian | | 5874(77.37) | 25.62±2.70 | 50.2±12.13 | US | NR |
| Zhu (2008) | (46) | Blood | Asian | | 26(54.2) | NR | 27-54 | US | NR |
| El-Derany (2020) | (47) | Serum | African | | 79(100) | 29±4.3 | 1.2±5.9 | Liver biopsy | ELISA |
| Kumar (2020) | (48) | Serum | Asian | | NR | 25.75±2.7 | 39.19±9.67 | US | latex agglutination assay |
| Ma (2020) | (49) | Plasma | Asian | | 36(34.3) | 28.56±3.87 | 52.51±9.09 | US | NR |
| Dallio (2021) | (50) | NR | European | | 13(26） | 29.09±3.1 | 45.2±15 | Liver biopsy | NR |
| Taniguchi (2021) | (51) | NR | Asian | | 3214(100) | NR | 51(24-90) | US | NR |

Abbreviation: NR, not reported; BMI, body mass index; US, ultrasonography examination; ELISA, enzyme-linked immunosorbent assay; TIIA, turbidimetric inhibition immunoassay.

**Supplementary Table 5**. Laboratory indicators of included studies.

| **Study (Year)** | **Reference** | | **AST (IU/mL)** | **ALT (IU/mL)** | **GGT (IU/mL)** | **TG (mg/dL）** | **LDL-C (mg/dL)** | **HDL-C (mg/dL)** | **HOMA-IR** |
| --- | --- | --- | --- | --- | --- | --- | --- | --- | --- |
| Abdel-Razik (2016) | (1) | 0.059(0.046-0.082) | | 0.071(0.056-0.089) | NR | NR | NR | NR | NR |
| Ajmera (2017) | (2) | 0.0667±0.0436 | | 0.0904±0.0594 | NR | 194±125 | NR | 42.6±11.4 | 6.7±6.5 |
| Akinkugbe (2017) | (3) | NR | | NR | NR | NR | NR | NR | NR |
| Alisi (2010) | (4) | 0.0532±0.0316 | | 0.0871±0.08 | 0.0253±0.0167 | 97.7±54.6 | NR | NR | 2.3±1.1 |
| Alrifai (2015) | (5) | 0.044±0.019 | | 0.067±0.033 | NR | NR | 115.7±31.1 | 45±11.8 | NR |
| Barretto (2020) | (6) | 0.02±0.006 | | 0.02±0.01 | 0.025±0.0143 | 97.5±49.26 | 91.5±27.63 | 39.35±9.407 | 3.5±1.778 |
| Ceccarelli (2015) | (7) | NR | | NR | NR | NR | NR | NR | NR |
| Chiang (2012) | (8) | NR | | NR | NR | 143±90 | 106±32 | 41±12 | NR |
| Choi (2009) | (9) | 0.0265±0.0123 | | 0.0338±0.0232 | 0.0352±0.0307 | 145.1±68.9 | NR | 47.6±10.6 | NR |
| Chunming (2015) | (10) | 0.07096±0.02263 | | 0.10632±0.03443 | NR | 41.22±27.9 | 50.04±17.64 | 27.18±4.32 | NR |
| El-Ashmawy (2019) | (11) | 0.0356±0.0201 | | 0.0449±0.0263 | 0.0532±0.0425 | 189.4±85.2 | 108.5±34.1 | 45.4±9.3 | 4.9±3.7 |
| García-Galiano (2007) | (12) | 0.027±0.0055 | | 0.038±0.0076 | NR | NR | NR | 51.10±1.8 | 12±2.7 |
| Holterman (2013) | (13) | 0.034±0.023 | | 0.04±0.031 | NR | 108±56 | NR | 34.8±9 | 10±8.9 |
| Hossain (2016) | (14) | NR | | NR | 0.036±0.013 | 177±88 | 130±36 | 35±7 | 4.03±1.39 |
| Hui (2004) | (15) | NR | | 0.091±0.007 | NR | NR | NR | NR | 5.5±0.4 |
| Khoury (2019) | (16) | 0.0496±0.0159 | | 0.0817±0.0305 | NR | 148.9±29 | 134.7±22.3 | 37.3±8.4 | 3.1±1 |
| Klisic (2018) | (17) | 0.02(0.017-0.024) | | 0.024(0.017-0.035) | 0.024(0.017-0.034) | NR | 55.26(44.82-70.02) | 19.8(15.84-23.76) | NR |
| Kogiso (2009) | (18) | 0.0204±0.0046 | | 0.0224±0.0042 | NR | 167.0±82.6 | NR | 49.0±9.0 | NR |
| Koh (2009) | (19) | 0.0338±0.0192 | | 0.0426±0.0247 | 0.0521±0.0419 | 186.7±83.4 | 111.5±33.7 | 47.8±8.8 | 4.6±3.6 |
| Koo (2020) | (20) | 0.055(0.035-0.075) | | 0.07(0.037-0.113) | 0.059(0.036-0.086) | 138(101-194) | NR | 44(37-53) | 4.24(2.77-6.47) |
| Koot (2013) | (21) | NR | | 0.0318±0.019 | NR | 17.64±9 | 45±12.24 | 19.44±4.5 | 3.9±2.5 |
| Kosmalski (2013) | (22) | 0.03365±0.03148 | | 0.04659±0.05187 | NR | 60.3±66.6 | 56.34±21.24 | 19.44±9.36 | 8.02±6.19 |
| Kuppan (2012) | (23) | 0.024±0.0126 | | 0.0295±0.023 | NR | NR | 121±33 | 41±8.4 | 3.1±2.0 |
| Lee (2017) | (24) | 0.021(0.019-0.024) | | 0.02(0.016-0.024) | 0.019(0.014-0.025) | 141.0±72.4 | 115.8±27.2 | 54.7±11.6 | 1.33(1.07-1.71) |
| Liang (2015) | (25) | NR | | NR | NR | 46.7622±29.178 | NR | NR | NR |
| Mahamid (2015) | (26) | NR | | NR | NR | NR | NR | NR | NR |
| Mikolasevic (2020) | (27) | 0.021(0.017-0.031) | | 0.024(0.018-0.04) | NR | 39.42±12.42 | 53.46±11.34 | 20.88±2.88 | 4.6(2.7-7.2) |
| Musso (2008) | (28) | 0.035±0.012 | | 0.078±0.021 | 0.097±0.089 | 24.12±12.42 | 66.42±12.96 | 22.86±3.78 | 3.78±2.27 |
| Nigam (2013) | (29) | NR | | NR | NR | 166.36±78.85 | 106.8±24.1 | 41.31±7.2 | NR |
| Ogawa (2013) | (30) | 0.0415±0.0171 | | 0.0543±0.0269 | NR | NR | NR | NR | 3.66±2.01 |
| Park (2004) | (31) | NR | | NR | NR | 195.7±11.11 | 130.2±2.64 | 48.5±0.92 | 2.13±0.057 |
| Perito (2017) | (32) | 0.0836±0.0529 | | 0.1435±0.0997 | NR | 170.4±94.5 | NR | 35.6±6.8 | 10.5±15.1 |
| Price (2017) | (33) | 0.024(0.021-0.032) | | 0.03(0.021-0.043) | NR | 148(109-217) | NR | NR | NR |
| Riquelme (2009) | (34) | NR | | ＞0.014 | NR | 171.9±88 | 139.1±40.4 | 45.6±9.8 | ＞2.16 |
| Seo (2013) | (35) | 0.026(0.019-0.034) | | 0.027(0.018-0.036) | 0.029(0.0185-0.0475) | NR | 108.6±28.5 | 53.3±10.2 | 1.52±0.03 |
| Shin (2011) | (36) | 0.0266±0.0106 | | 0.0339±0.0163 | NR | 156(115-241) | 91.2±30.8 | 46.5±12.5 | 3.9±2.3 |
| Shoji (2016) | (37) | 0.054(0.027-0.185) | | 0.053(0.008-0.194) | NR | NR | NR | NR | NR |
| Simon (2018) | (38) | NR | | NR | NR | 182±158.7 | 115.7±31.1 | 44.6±12 | 2.88±2.1 |
| Sung (2009) | (39) | NR | | NR | NR | 32.94(32.4-33.66) | 55.08(54.54-55.62) | 21.24(21.06-21.42) | NR |
| Tabuchi (2010) | (40) | 0.025±0.009 | | 0.035±0.019 | NR | 147±86 | 139±20 | 48±10 | NR |
| Wang (2016) | (41) | NR | | NR | NR | 24.48±15.12 | 45.54±10.98 | 23.4±5.22 | NR |
| Wu (2015) | (42) | 0.0565±0.0167 | | 0.0595±0.0208 | NR | 64.8±14.4 | 66.6±7.2 | 30.6±16.2 | 3.7±0.5 |
| Yeniova (2014) | (43) | 0.032±0.0013 | | 0.04288±0.0028 | 0.0384±0.0022 | 160±7 | 117.3±2.6 | 46.15±1.17 | 3.74±0.21 |
| Yoneda (2007) | (44) | 53±35.5 | | 80.6±60.0 | NR | 172.6±98.5 | 129.3±38.5 | 47.9±11.5 | 4.07±3.73 |
| Yu (2018) | (45) | 0.024(0.02-0.029) | | 0.027(0.02-0.039) | NR | 31.5(22.86-43.74) | 52.74(44.1-61.38) | 19.44(16.92-22.14) | 2.85(2.09-3.87) |
| Zhu (2008) | (46) | NR | | NR | NR | NR | NR | NR | NR |
| El-Derany (2020) | (47) | 0.053±0.035 | | 0.048±0.021 | NR | NR | NR | NR | 3±0.9 |
| Kumar (2020) | (48) | 0.0246±0.0068 | | 0.0289±0.0101 | NR | NR | NR | NR | NR |
| Ma (2020) | (49) | 0.0237(0.02055-0.0318) | | 0.03125(0.02193-0.04295) | NR | NR | 54.72±12.78 | 21.6±3.96 | 1.34±0.56 |
| Dallio (2021) | (50) | 0.034±0.02 | | 0.057±0.028 | 0.059±0.031 | NR | NR | NR | 6.59±3.26 |
| Taniguchi (2021) | (51) | NR | | NR | NR | NR | NR | NR | NR |

The statistics presented are means ± SD or medians.

Abbreviation: NR, not reported. AST, aspartate aminotransferase; ALT, alanine aminotransferase; GGT, gamma-glutamyl transpeptidase; TG, triglycerides; LDL-C, low density lipoprotein cholesterol; HDL-C, high-density lipoprotein cholesterol; HOMA-IR, homeostasis model assessment of insulin resistance index.

1. Abdel-Razik A, Mousa N, Shabana W, Refaey M, ElMahdy Y, Elhelaly R, et al. A novel model using mean platelet volume and neutrophil to lymphocyte ratio as a marker of nonalcoholic steatohepatitis in NAFLD patients: multicentric study. Eur J Gastroenterol Hepatol. 2016;28(1):e1-9.

2. Ajmera V, Perito ER, Bass NM, Terrault NA, Yates KP, Gill R, et al. Novel plasma biomarkers associated with liver disease severity in adults with nonalcoholic fatty liver disease. Hepatology. 2017;65(1):65-77.

3. Akinkugbe AA, Avery CL, Barritt AS, Cole SR, Lerch M, Mayerle J, et al. Do Genetic Markers of Inflammation Modify the Relationship between Periodontitis and Nonalcoholic Fatty Liver Disease? Findings from the SHIP Study. J Dent Res. 2017;96(12):1392-9.

4. Alisi A, Manco M, Devito R, Piemonte F, Nobili V. Endotoxin and plasminogen activator inhibitor-1 serum levels associated with nonalcoholic steatohepatitis in children. J Pediatr Gastroenterol Nutr. 2010;50(6):645-9.

5. Al Rifai M, Silverman MG, Nasir K, Budoff MJ, Blankstein R, Szklo M, et al. The association of nonalcoholic fatty liver disease, obesity, and metabolic syndrome, with systemic inflammation and subclinical atherosclerosis: the Multi-Ethnic Study of Atherosclerosis (MESA). Atherosclerosis. 2015;239(2):629-33.

6. Barretto JR, Boa-Sorte N, Vinhaes CL, Malta-Santos H, Reboucas-Silva J, Ramos CF, et al. Heightened Plasma Levels of Transforming Growth Factor Beta (TGF-beta) and Increased Degree of Systemic Biochemical Perturbation Characterizes Hepatic Steatosis in Overweight Pediatric Patients: A Cross-Sectional Study. Nutrients. 2020;12(6).

7. Ceccarelli S, Panera N, Mina M, Gnani D, De Stefanis C, Crudele A, et al. LPS-induced TNF-α factor mediates pro-inflammatory and pro-fibrogenic pattern in non-alcoholic fatty liver disease. Oncotarget. 2015;6(39):41434-52.

8. Chiang CH, Huang PH, Chung FP, Chen ZY, Leu HB, Huang CC, et al. Decreased circulating endothelial progenitor cell levels and function in patients with nonalcoholic fatty liver disease. PLoS One. 2012;7(2):e31799.

9. Choi S-Y, Kim D, Kim HJ, Kang JH, Chung SJ, Park MJ, et al. The Relation Between Non-Alcoholic Fatty Liver Disease and the Risk of Coronary Heart Disease in Koreans. The American Journal of Gastroenterology. 2009;104(8):1953-60.

10. Chunming L, Jianhui S, Hongguang Z, Chunwu Q, Xiaoyun H, Lijun Y, et al. The development of a clinical score for the prediction of nonalcoholic steatohepatitis in patients with nonalcoholic fatty liver disease using routine parameters. Turk J Gastroenterol. 2015;26(5):408-16.

11. El-Ashmawy HM, Ahmed AM. Serum fetuin-B level is an independent marker for nonalcoholic fatty liver disease in patients with type 2 diabetes. Eur J Gastroenterol Hepatol. 2019;31(7):859-64.

12. García-Galiano D, Sánchez-Garrido MA, Espejo I, Montero JL, Costán G, Marchal T, et al. IL-6 and IGF-1 are independent prognostic factors of liver steatosis and non-alcoholic steatohepatitis in morbidly obese patients. Obesity surgery. 2007;17(4):493-503.

13. Holterman AX, Guzman G, Fantuzzi G, Wang H, Aigner K, Browne A, et al. Nonalcoholic fatty liver disease in severely obese adolescent and adult patients. Obesity (Silver Spring). 2013;21(3):591-7.

14. Hossain IA, Akter S, Bhuiyan FR, Shah MR, Rahman MK, Ali L. Subclinical inflammation in relation to insulin resistance in prediabetic subjects with nonalcoholic fatty liver disease. BMC Res Notes. 2016;9:266.

15. Hui JM, Hodge A, Farrell GC, Kench JG, Kriketos A, George J. Beyond insulin resistance in NASH: TNF-alpha or adiponectin? Hepatology. 2004;40(1):46-54.

16. Khoury T, Mari A, Nseir W, Kadah A, Sbeit W, Mahamid M. Neutrophil-to-lymphocyte ratio is independently associated with inflammatory activity and fibrosis grade in nonalcoholic fatty liver disease. Eur J Gastroenterol Hepatol. 2019;31(9):1110-5.

17. Klisic A, Isakovic A, Kocic G, Kavaric N, Jovanovic M, Zvrko E, et al. Relationship between Oxidative Stress, Inflammation and Dyslipidemia with Fatty Liver Index in Patients with Type 2 Diabetes Mellitus. Exp Clin Endocrinol Diabetes. 2018;126(6):371-8.

18. Kogiso T, Moriyoshi Y, Shimizu S, Nagahara H, Shiratori K. High-sensitivity C-reactive protein as a serum predictor of nonalcoholic fatty liver disease based on the Akaike Information Criterion scoring system in the general Japanese population. J Gastroenterol. 2009;44(4):313-21.

19. Koh JH, Shin YG, Nam SM, Lee MY, Chung CH, Shin JY. Serum adipocyte fatty acid-binding protein levels are associated with nonalcoholic fatty liver disease in type 2 diabetic patients. Diabetes Care. 2009;32(1):147-52.

20. Koo BK, Joo SK, Kim D, Lee S, Bae JM, Park JH, et al. Development and Validation of a Scoring System, Based on Genetic and Clinical Factors, to Determine Risk of Steatohepatitis in Asian Patients with Nonalcoholic Fatty Liver Disease. Clin Gastroenterol Hepatol. 2020;18(11):2592-9 e10.

21. Koot BG, van der Baan-Slootweg OH, Bohte AE, Nederveen AJ, van Werven JR, Tamminga-Smeulders CL, et al. Accuracy of prediction scores and novel biomarkers for predicting nonalcoholic fatty liver disease in obese children. Obesity (Silver Spring). 2013;21(3):583-90.

22. Kosmalski M, Kasznicki J, Drzewoski J. Relationship between ultrasound features of nonalcoholic fatty liver disease and cardiometabolic risk factors in patients with newly diagnosed type 2 diabetes. Polskie Archiwum Medycyny Wewnetrznej. 2013;123(9):436-42.

23. Kuppan G, Anjana RM, Deepa M, Paramasivam P, Chandrakumar S, Kaliyaperumal V, et al. Inflammatory markers in relation to nonalcoholic fatty liver disease in urban South Indians. Diabetes Technol Ther. 2012;14(2):152-8.

24. Lee J, Yoon K, Ryu S, Chang Y, Kim HR. High-normal levels of hs-CRP predict the development of non-alcoholic fatty liver in healthy men. PLoS One. 2017;12(2):e0172666.

25. Liang C-C, Ding Y-N, Ji W-J, Shi J, Yeer N-E. Risk factors for nonalcoholic fatty liver disease in Uygur people in Urumqi. World Chinese Journal of Digestology. 2015;23(25).

26. Mahamid M, Kalman P, Wengrover D. P1081 : Hyperplastic colonic polyps link to nonalcoholic steatohepatitis and vitamin D deficiency. Journal of Hepatology. 2015;62.

27. Mikolasevic I, Domislovic V, Turk Wensveen T, Delija B, Klapan M, Juric T, et al. Screening for nonalcoholic fatty liver disease in patients with type 2 diabetes mellitus using transient elastography - a prospective, cross sectional study. Eur J Intern Med. 2020;82:68-75.

28. Musso G, Gambino R, Bo S, Uberti B, Biroli G, Pagano G, et al. Should nonalcoholic fatty liver disease be included in the definition of metabolic syndrome? A cross-sectional comparison with Adult Treatment Panel III criteria in nonobese nondiabetic subjects. Diabetes Care. 2008;31(3):562-8.

29. Nigam P, Bhatt SP, Misra A, Vaidya M, Dasgupta J, Chadha DS. Non-alcoholic fatty liver disease is closely associated with sub-clinical inflammation: a case-control study on Asian Indians in North India. PLoS One. 2013;8(1):e49286.

30. Ogawa Y, Imajo K, Yoneda M, Kessoku T, Tomeno W, Shinohara Y, et al. Soluble CD14 levels reflect liver inflammation in patients with nonalcoholic steatohepatitis. PLoS One. 2013;8(6):e65211.

31. Park SH, Kim BI, Yun JW, Kim JW, Park DI, Cho YK, et al. Insulin resistance and C-reactive protein as independent risk factors for non-alcoholic fatty liver disease in non-obese Asian men. Journal of gastroenterology and hepatology. 2004;19(6):694-8.

32. Perito ER, Ajmera V, Bass NM, Rosenthal P, Lavine JE, Schwimmer JB, et al. Association Between Cytokines and Liver Histology in Children with Nonalcoholic Fatty Liver Disease. Hepatol Commun. 2017;1(7):609-22.

33. Price JC, Wang R, Seaberg EC, Budoff MJ, Kingsley LA, Palella FJ, et al. The Association of Inflammatory Markers With Nonalcoholic Fatty Liver Disease Differs by Human Immunodeficiency Virus Serostatus. Open Forum Infect Dis. 2017;4(3):ofx153.

34. Riquelme A, Arrese M, Soza A, Morales A, Baudrand R, Perez-Ayuso RM, et al. Non-alcoholic fatty liver disease and its association with obesity, insulin resistance and increased serum levels of C-reactive protein in Hispanics. Liver Int. 2009;29(1):82-8.

35. Seo YY, Cho YK, Bae JC, Seo MH, Park SE, Rhee EJ, et al. Tumor Necrosis Factor-alpha as a Predictor for the Development of Nonalcoholic Fatty Liver Disease: A 4-Year Follow-Up Study. Endocrinol Metab (Seoul). 2013;28(1):41-5.

36. Shin JY, Kim SK, Lee MY, Kim HS, Ye BI, Shin YG, et al. Serum sex hormone-binding globulin levels are independently associated with nonalcoholic fatty liver disease in people with type 2 diabetes. Diabetes Res Clin Pract. 2011;94(1):156-62.

37. Shoji H, Yoshio S, Mano Y, Kumagai E, Sugiyama M, Korenaga M, et al. Interleukin-34 as a fibroblast-derived marker of liver fibrosis in patients with non-alcoholic fatty liver disease. Sci Rep. 2016;6:28814.

38. Simon TG, Trejo MEP, McClelland R, Bradley R, Blaha MJ, Zeb I, et al. Circulating Interleukin-6 is a biomarker for coronary atherosclerosis in nonalcoholic fatty liver disease: Results from the Multi-Ethnic Study of Atherosclerosis. Int J Cardiol. 2018;259:198-204.

39. Sung KC, Ryan MC, Wilson AM. The severity of nonalcoholic fatty liver disease is associated with increased cardiovascular risk in a large cohort of non-obese Asian subjects. Atherosclerosis. 2009;203(2):581-6.

40. Tabuchi M, Tomioka K, Kawakami T, Murakami Y, Hiramatsu M, Itoshima T, et al. Serum cytokeratin 18 M30 antigen level and its correlation with nutritional parameters in middle-aged Japanese males with nonalcoholic fatty liver disease (NAFLD). Journal of nutritional science and vitaminology. 2010;56(5):271-8.

41. Wang LR, Liu WY, Wu SJ, Zhu GQ, Lin YQ, Braddock M, et al. Parabolic relationship between sex-specific serum high sensitive C reactive protein and non-alcoholic fatty liver disease in Chinese adults: a large population-based study. Oncotarget. 2016;7(12):14241-50.

42. Wu PB, Deng YZ, Shu YX, Tan SY, Li M, Fang G. Increased plasma CgA levels associated with nonalcoholic fatty liver disease. Turk J Gastroenterol. 2015;26(5):404-7.

43. Yeniova AO, Küçükazman M, Ata N, Dal K, Kefeli A, Başyiğit S, et al. High-sensitivity C-reactive protein is a strong predictor of non-alcoholic fatty liver disease. Hepato-gastroenterology. 2014;61(130):422-5.

44. Yoneda M, Mawatari H, Fujita K, Iida H, Yonemitsu K, Kato S, et al. High-sensitivity C-reactive protein is an independent clinical feature of nonalcoholic steatohepatitis (NASH) and also of the severity of fibrosis in NASH. J Gastroenterol. 2007;42(7):573-82.

45. Yu YY, Cai JT, Song ZY, Tong YL, Wang JH. The associations among Helicobacter pylori infection, white blood cell count and nonalcoholic fatty liver disease in a large Chinese population. Medicine (Baltimore). 2018;97(46):e13271.

46. Zhu QX, Deng CS. Detection and significance of serum TNF-α, TGF-β1 and hs-CRP in patients with non-alcoholic fatty liver disease. World Chinese Journal of Digestology. 2008;16(34):3910-2.

47. El-Derany MO. Polymorphisms in Interleukin 13 Signaling and Interacting Genes Predict Advanced Fibrosis and Hepatocellular Carcinoma Development in Non-Alcoholic Steatohepatitis. Biology. 2020;9(4).

48. Kumar R, Porwal YC, Dev N, Kumar P, Chakravarthy S, Kumawat A. Association of high-sensitivity C-reactive protein (hs-CRP) with non-alcoholic fatty liver disease (NAFLD) in Asian Indians: A cross-sectional study. Journal of family medicine and primary care. 2020;9(1):390-4.

49. Ma C, Liu Y, He S, Zeng J, Li P, Ma C, et al. Association Between Leukocyte Mitochondrial DNA Copy Number and Non-alcoholic Fatty Liver Disease in a Chinese Population Is Mediated by 8-Oxo-2'-Deoxyguanosine. Frontiers in medicine. 2020;7:536.

50. Dallio M, Masarone M, Romeo M, Tuccillo C, Morisco F, Persico M, et al. PNPLA3, TM6SF2, and MBOAT7 Influence on Nutraceutical Therapy Response for Non-alcoholic Fatty Liver Disease: A Randomized Controlled Trial. Frontiers in medicine. 2021;8:734847.

51. Taniguchi H, Iwasaki Y, Aimi M, Matsushita H. Relationship between fatty liver and high-sensitivity C-reactive protein. United European Gastroenterology Journal. 2021;9(SUPPL 8):657-8.

52. Mehta R, Afendy A, Baranova A, Goodman Z, Younossi ZM. Differential expression of inflammasomes components in patients with non-alcoholic fatty liver disease (NAFLD). Hepatology. 2012;56:872A.

53. Schlattjan M, Wree A, Bechmann LP, Gerken G, Canbay A. Visceral adipose tissue affects liver injury of NAFLD and NASH patients. Journal of Hepatology. 2014;60(1):S158.

54. Adams LA, Wree A, Melton P, Jeffrey GP, Ching H, De Boer B, et al. Serum marker of inflammasome activity correlates with liver injury in nonalcoholic fatty liver disease and is influenced by genetic polymorphisms. Hepatology. 2015;62:1273A.

55. Li Y, Xiong F, Xu W, Li X, Liu SD. High level angiotensin II and activated NLRP3 inflammasome are associated with nonalcoholic fatty liver disease. Journal of Digestive Diseases. 2015;16:1-2.

56. Mitsuyoshi H, Yasui K, Hara T, Taketani H, Ishiba H, Okajima A, et al. Hepatic NOD-like receptors, pyrin domain-containing 3 (NLRP3) inflammasome activation is associated with histological severity in patients with nonalcoholic fatty liver disease. Hepatology. 2015;62:647A.

57. Sowa JP, Schlattjan M, Gerken G, Canbay A. Adipocyte hypertrophy and inflammation could affect liver injury in NAFLD by alternative mechanisms. United European Gastroenterology Journal. 2015;3(5):A503-A4.

58. Kang SH, Yeon JE, Je JH, Lee YS, Yoo YJ, Suh SJ, et al. Nucleotide binding and oligomerization domain like receptors (NLR) inflammasomes in patients with non-alcoholic fatty liver disease (NAFLD). Hepatology. 2016;64(1):792A.

59. Neuman MG, Cohen LB, Maor Y, Hilzenrat N. Biomarkers to differentiate alcoholic and non-acoholic steatohepatitis, hepatitis C, and benign asymptomatic transaminase elevation. Alcoholism: Clinical and Experimental Research. 2016;40:95A.

60. Mitsuyoshi H, Yasui K, Hara T, Taketani H, Ishiba H, Okajima A, et al. Hepatic nucleotide binding oligomerization domain-like receptors pyrin domain-containing 3 inflammasomes are associated with the histologic severity of non-alcoholic fatty liver disease. Hepatology research : the official journal of the Japan Society of Hepatology. 2017;47(13):1459-68.

61. Li Y, Xiong F, Xu W, Liu S. Increased Serum Angiotensin II Is a Risk Factor of Nonalcoholic Fatty Liver Disease: A Prospective Pilot Study. Gastroenterology Research and Practice. 2019;2019.

62. Rau M, Krawczyk M, Paukstat K, Lammert F, Geier A. The NLRP3 inflammasome rs10754558 is associated with increased serum ALT activities: Analysis of a prospective NAFLD cohort. Zeitschrift fur Gastroenterologie. 2019;57(1):e50.

63. Cyr B, Keane RW, Vaccari JPR. Asc, il‐18 and galectin‐3 as biomarkers of non‐alcoholic steatohepatitis: A proof of concept study. International Journal of Molecular Sciences. 2020;21(22):1-13.

64. Sim JH, Sherman JB, Fitch KV, Looby SE, Robinson JA, Lu M, et al. Il-18 is associated with hepatosteatosis and higher liver enzymes in people with HIV. Topics in Antiviral Medicine. 2020;28(1):196.

65. Unamuno X, Gómez Ambrosi J, Ramírez B, Rodríguez A, Becerril S, Valentí V, et al. Obesity triggers NLRP3-dependent adipose tissue low-grade inflammation and extracellular matrix remodelling. Obesity Reviews. 2020;21(SUPPL 1).

66. Quezada N, Valencia I, Torres J, Maturana G, Cerda J, Arab JP, et al. Insulin resistance and liver histopathology in metabolically unhealthy subjects do not correlate with the hepatic abundance of NLRP3 inflammasome nor circulating IL-1 beta levels. BMJ open diabetes research & care. 2021;9(1).

67. Unamuno X, Gómez-Ambrosi J, Ramírez B, Rodríguez A, Becerril S, Valentí V, et al. NLRP3 inflammasome as a mediator of inflammation and extracellular matrix remodelling in obesity-associated non-alcoholic fatty liver disease. European Journal of Clinical Investigation. 2020;50(SUPPL 1):13-4.

68. Sim JH, Sherman JB, Stanley TL, Corey KE, Fitch KV, Looby SE, et al. Pro-Inflammatory Interleukin-18 is Associated with Hepatic Steatosis and Elevated Liver Enzymes in People with HIV Monoinfection. AIDS research and human retroviruses. 2021;37(5):385-90.

69. Unamuno X, Gómez-Ambrosi J, Ramírez B, Rodríguez A, Becerril S, Valentí V, et al. NLRP3 inflammasome blockade reduces adipose tissue inflammation and extracellular matrix remodeling. Cellular & molecular immunology. 2021;18(4):1045-57.

70. Zhu X, Lin X, Zhang P, Liu Y, Ling W, Guo H. Upregulated NLRP3 inflammasome activation is attenuated by anthocyanins in patients with nonalcoholic fatty liver disease: A case-control and an intervention study. Clinics and research in hepatology and gastroenterology. 2022;46(4).

**Supplementary Table 6a.** Subgroup analyses of the association between inflammatory cytokines and NAFLD.

| **Inflammatory**  **cytokines** | **Subgroup** | **No. of studies** | **OR（95%CI）** | ***P* value** | ***P* for heterogeneity** | ***I²*（%）** |
| --- | --- | --- | --- | --- | --- | --- |
| **IL-2** | **Overall** | 5 | 1.02(0.98, 1.07) | 0.388 | 0.144 | 41.6 |
|  | **Race** |  |  |  |  |  |
|  | Asian | 0 | - | - | - | - |
|  | Caucasian | 5 | 1.02(0.98, 1.07) | 0.388 | 0.144 | 41.6 |
|  | Others | 0 | - | - | - | - |
|  | **Mean age** |  |  |  |  |  |
|  | ＜18 | 1 | 1.03(0.90, 1.17) | 0.659 | - | - |
|  | 18-60 | 3 | 1.00(0.95, 1.06) | 0.962 | 0.090 | 58.6 |
|  | ≥60 | 1 | 1.09(0.98, 1.21) | 0.109 | - | - |
|  | Missing | - | - | - | - | - |
|  | **BMI** |  |  |  |  |  |
|  | ＜30 | 0 | - | - | - | - |
|  | ≥30 | 4 | 1.02(0.97, 1.07) | 0.448 | 0.078 | 56.0 |
|  | Missing | 1 | 1.03(0.90, 1.17) | 0.659 | - | - |
|  | **Sample** |  |  |  |  |  |
|  | Serum | 1 | 1.09(0.98, 1.21) | 0.109 | - | - |
|  | Plasma | 4 | 1.01(0.96, 1.06) | 0.835 | 0.173 | 39.8 |
|  | Blood | 0 | - | - | - | - |
|  | Missing | 0 | - | - | - | - |
|  | **Diagnose** |  |  |  |  |  |
|  | Liver biopsy | 4 | 1.01(0.96, 1.06) | 0.835 | 0.173 | 39.8 |
|  | US | 0 | - | - | - | - |
|  | CT | 1 | 1.09(0.98, 1.21) | 0.109 | - | - |
|  | **Measurement** |  |  |  |  |  |
|  | ELISA | 2 | 1.07(0.98, 1.16) | 0.127 | 0.510 | 0.0 |
|  | Others | 3 | 1.00(0.95, 1.06) | 0.962 | 0.090 | 58.6 |
|  |  |  |  |  |  |  |
| **IL-4** | **Overall** | 4 | 1.01(0.96,1.05) | 0.781 | 0.530 | 0.0 |
|  | **Race** |  |  |  |  |  |
|  | Asian | 0 | - | - | - | - |
|  | Caucasian | 4 | 1.01(0.96,1.05) | 0.781 | 0.530 | 0.0 |
|  | Others | 0 | - | - | - | - |
|  | **Mean age** |  |  |  |  |  |
|  | ＜18 | 1 | 1.01(0.88,1.16) | 0.888 | - | - |
|  | 18-60 | 3 | 1.01(0.96,1.05) | 0.806 | 0.332 | 9.4 |
|  | ≥60 | 0 | - | - | - | - |
|  | Missing | 0 | - | - | - | - |
|  | **BMI** |  |  |  |  |  |
|  | ＜30 | 0 | - | - | - | - |
|  | ≥30 | 3 | 1.01(0.96,1.05) | 0.806 | 0.332 | 9.4 |
|  | Missing | 1 | 1.01(0.88,1.16) | 0.888 | - | - |
|  | **Sample** |  |  |  |  |  |
|  | Serum | 0 | - | - | - | - |
|  | Plasma | 4 | 1.01(0.96,1.05) | 0.781 | 0.530 | 0.0 |
|  | Blood | 0 | - | - | - | - |
|  | Missing | 0 | - | - | - | - |
|  | **Diagnose** |  |  |  |  |  |
|  | Liver biopsy | 4 | 1.01(0.96,1.05) | 0.781 | 0.530 | 0.0 |
|  | US | 0 | - | - | - | - |
|  | CT | 0 | - | - | - | - |
|  | **Measurement** |  |  |  |  |  |
|  | ELISA | 1 | 1.01(0.88,1.16) | 0.888 | - | - |
|  | Others | 3 | 1.01(0.96,1.05) | 0.806 | 0.332 | 9.4 |
|  |  |  |  |  |  |  |
| **IL-5** | **Overall** | 4 | 0.98(0.94,1.03) | 0.473 | 0.736 | 0.0 |
|  | **Race** |  |  |  |  |  |
|  | Asian | 0 | - | - | - | - |
|  | Caucasian | 4 | 0.98(0.94,1.03) | 0.473 | 0.736 | 0.0 |
|  | Others | 0 | - | - | - | - |
|  | **Mean age** |  |  |  |  |  |
|  | ＜18 | 1 | 1.04(0.91,1.19) | 0.567 | - | - |
|  | 18-60 | 3 | 0.98(0.93,1.02) | 0.336 | 0.766 | 0.0 |
|  | ≥60 | 0 | - | - | - | - |
|  | Missing | 0 | - | - | - | - |
|  | **BMI** |  |  |  |  |  |
|  | ＜30 | 0 | - | - | - | - |
|  | ≥30 | 3 | 0.98(0.93,1.02) | 0.336 | 0.766 | 0.0 |
|  | Missing | 1 | 1.04(0.91,1.19) | 0.567 | - | - |
|  | **Sample** |  |  |  |  |  |
|  | Serum | 0 | - | - | - | - |
|  | Plasma | 4 | 0.98(0.94,1.03) | 0.473 | 0.736 | 0.0 |
|  | Blood | 0 | - | - | - | - |
|  | Missing | 0 | - | - | - | - |
|  | **Diagnose** |  |  |  | - | - |
|  | Liver biopsy | 4 | 0.98(0.94,1.03) | 0.473 | 0.736 | 0.0 |
|  | US | 0 | - | - | - | - |
|  | CT | 0 | - | - | - | - |
|  | **Measurement** |  |  |  |  |  |
|  | ELISA | 1 | 1.04(0.91,1.19) | 0.567 | - | - |
|  | Others | 3 | 0.98(0.93,1.02) | 0.336 | 0.766 | 0.0 |
|  |  |  |  |  |  |  |
| **IL-7** | **Overall** | 4 | 1.02(0.97,1.06) | 0.453 | 0.301 | 17.9 |
|  | **Race** |  |  |  |  |  |
|  | Asian | 0 | - | - | - | - |
|  | Caucasian | 4 | 1.02(0.97,1.06) | 0.453 | 0.301 | 17.9 |
|  | Others | 0 | - | - | - | - |
|  | **Mean age** |  |  |  |  |  |
|  | ＜18 | 1 | 0.96(0.82,1.12) | 0.608 | - | - |
|  | 18-60 | 3 | 1.02(0.98,1.07) | 0.353 | 0.213 | 35.3 |
|  | ≥60 | 0 | - | - | - | - |
|  | Missing | 0 | - | - | - | - |
|  | **BMI** |  |  |  |  |  |
|  | ＜30 | 0 | - | - | - | - |
|  | ≥30 | 3 | 1.02(0.98,1.07) | 0.353 | 0.213 | 35.3 |
|  | Missing | 1 | 0.96(0.82,1.12) | 0.608 | - | - |
|  | **Sample** |  |  |  |  |  |
|  | Serum | 0 | - | - | - | - |
|  | Plasma | 4 | 1.02(0.97,1.06) | 0.453 | 0.301 | 17.9 |
|  | Blood | 0 | - | - | - | - |
|  | Missing | 0 | - | - | - | - |
|  | **Diagnose** |  |  |  |  |  |
|  | Liver biopsy | 4 | 1.02(0.97,1.06) | 0.453 | 0.301 | 17.9 |
|  | US | 0 | - | - | - | - |
|  | CT | 0 | - | - | - | - |
|  | **Measurement** |  |  |  |  |  |
|  | ELISA | 1 | 0.96(0.82,1.12) | 0.608 | - | - |
|  | Others | 3 | 1.02(0.98,1.07) | 0.353 | 0.213 | 35.3 |
|  |  |  |  |  |  |  |
| **IL-8** | **Overall** | 4 | 1.22(0.99,1.51) | 0.061 | 0.000 | 90.9 |
|  | **Race** |  |  |  |  |  |
|  | Asian | 0 | - | - | - | - |
|  | Caucasian | 4 | 1.22(0.99,1.51) | 0.061 | 0.000 | 90.9 |
|  | Others | 0 | - | - | - | - |
|  | **Mean age** |  |  |  |  |  |
|  | ＜18 | 1 | 1.11(0.97,1.27) | 0.129 | - | - |
|  | 18-60 | 3 | 1.27(0.94,1.72) | 0.117 | 0.000 | 93.9 |
|  | ≥60 | 0 | - | - | - | - |
|  | Missing | 0 | - | - | - | - |
|  | **BMI** |  |  |  |  |  |
|  | ＜30 | 0 | - | - | - | - |
|  | ≥30 | 3 | 1.27(0.94,1.72) | 0.117 | 0.000 | 93.9 |
|  | Missing | 1 | 1.11(0.97,1.27) | 0.129 | - | - |
|  | **Sample** |  |  |  |  |  |
|  | Serum | 0 | - | - | - | - |
|  | Plasma | 4 | 1.22(0.99,1.51) | 0.061 | 0.000 | 90.9 |
|  | Blood | 0 | - | - | - | - |
|  | Missing | 0 | - | - | - | - |
|  | **Diagnose** |  |  |  |  |  |
|  | Liver biopsy | 4 | 1.22(0.99,1.51) | 0.061 | 0.000 | 90.9 |
|  | US | 0 | - | - | - | - |
|  | CT | 0 | - | - | - | - |
|  | **Measurement** |  |  |  |  |  |
|  | ELISA | 1 | 1.11(0.97,1.27) | 0.129 | - | - |
|  | Others | 3 | 1.27(0.94,1.72) | 0.117 | 0.000 | 93.9 |
|  |  |  |  |  |  |  |
| **IL-10** | **Overall** | 4 | 1.01(1.00-1.01) | 0.154 | 0.591 | 0.0 |
|  | **Race** |  |  |  |  |  |
|  | Asian | 0 | - | - | - | - |
|  | Caucasian | 4 | 1.01(1.00-1.01) | 0.154 | 0.591 | 0.0 |
|  | Others | 0 | - | - | - | - |
|  | **Mean age** |  |  |  |  |  |
|  | ＜18 | 1 | 0.98(0.83,1.15) | 0.808 | - | - |
|  | 18-60 | 3 | 1.01(1.00,1.01) | 0.151 | 0.403 | 0.0 |
|  | ≥60 | 0 | - |  | - | - |
|  | Missing | 0 | - |  | - | - |
|  | **BMI** |  |  |  |  |  |
|  | ＜30 | 0 | - | - | - | - |
|  | ≥30 | 3 | 1.01(1.00,1.01) | 0.151 | 0.403 | 0.0 |
|  | Missing | 1 | 0.98(0.83,1.15) | 0.808 | - | - |
|  | **Sample** |  |  |  |  |  |
|  | Serum | 0 | - | - | - | - |
|  | Plasma | 4 | 1.01(1.00-1.01) | 0.154 | 0.591 | 0.0 |
|  | Blood | 0 | - | - | - | - |
|  | Missing | 0 | - | - | - | - |
|  | **Diagnose** |  |  |  |  |  |
|  | Liver biopsy | 4 | 1.01(1.00-1.01) | 0.154 | 0.591 | 0.0 |
|  | US | 0 | - | - | - | - |
|  | CT | 0 | - | - | - | - |
|  | **Measurement** |  |  |  |  |  |
|  | ELISA | 1 | 0.98(0.83,1.15) | 0.808 | - | - |
|  | Others | 3 | 1.01(1.00,1.01) | 0.151 | 0.403 | 0.0 |
|  |  |  |  |  |  |  |
| **IL-12** | **Overall** | 3 | 0.99(0.94,1.04) | 0.654 | 0.280 | 21.5 |
|  | **Race** |  |  |  |  |  |
|  | Asian | 0 | - | - | - | - |
|  | Caucasian | 3 | 0.99(0.94,1.04) | 0.654 | 0.280 | 21.5 |
|  | Others | 0 | - | - | - | - |
|  | **Mean age** |  |  |  |  |  |
|  | ＜18 | 0 | - | - | - | - |
|  | 18-60 | 3 | 0.99(0.94,1.04) | 0.654 | 0.280 | 21.5 |
|  | ≥60 | 0 | - | - | - | - |
|  | Missing | 0 | - | - | - | - |
|  | **BMI** |  |  |  |  |  |
|  | ＜30 | 0 | - | - | - | - |
|  | ≥30 | 3 | 0.99(0.94,1.04) | 0.654 | 0.280 | 21.5 |
|  | Missing | 0 | - | - | - | - |
|  | **Sample** |  |  |  |  |  |
|  | Serum | 0 | - | - | - | - |
|  | Plasma | 3 | 0.99(0.94,1.04) | 0.654 | 0.280 | 21.5 |
|  | Blood | 0 | - | - | - | - |
|  | Missing | 0 | - | - | - | - |
|  | **Diagnose** |  |  |  |  |  |
|  | Liver biopsy | 3 | 0.99(0.94,1.04) | 0.654 | 0.280 | 21.5 |
|  | US | 0 | - | - | - | - |
|  | CT | 0 | - | - | - | - |
|  | **Measurement** |  |  |  |  |  |
|  | ELISA | 0 | - | - | - | - |
|  | Others | 3 | 0.99(0.94,1.04) | 0.654 | 0.280 | 21.5 |

Abbreviation: No. of studies, number of studies; OR (95%CI), odds ratios (95% confidence intervals); US, ultrasonography examination; CT, computed tomography.

**Supplementary Table 6b.** Subgroup analyses of the association between inflammatory cytokines and NAFLD.

| **Inflammatory**  **cytokines** | **Subgroup** | **No. of studies** | **OR（95%CI）** | ***P* value** | ***P* for heterogeneity** | ***I²*（%）** |
| --- | --- | --- | --- | --- | --- | --- |
| **TGF-β** | **Overall** | 10 | 1.05(0.98,1.12) | 0.136 | 0.001 | 67.8 |
|  | **Race** |  |  |  |  |  |
|  | Asian | 1 | 1.92(1.28,2.88) | 0.002 | - | - |
|  | Caucasian | 9 | 1.04(0.98,1.09) | 0.217 | 0.015 | 58.0 |
|  | Others | 0 | - | - | - | - |
|  | **Mean age** |  |  |  |  |  |
|  | ＜18 | 3 | 1.20(0.81,1.79) | 0.360 | 0.000 | 87.5 |
|  | 18-60 | 7 | 1.05(1.01,1.10) | 0.027 | 0.001 | 39.9 |
|  | ≥60 | 0 | - | - | - | - |
|  | Missing | 0 | - | - | - | - |
|  | **BMI** |  |  |  |  |  |
|  | ＜30 | 0 | - | - | - | - |
|  | ≥30 | 6 | 1.04(1.01,1.08) | 0.014 | 0.934 | 0.0 |
|  | Missing | 4 | 1.38(0.93,2.05) | 0.113 | 0.000 | 88.6 |
|  | **Sample** |  |  |  |  |  |
|  | Serum | 1 | 1.92(1.28,2.88) | 0.002 | - | - |
|  | Plasma | 9 | 1.04(0.98,1.09) | 0.217 | 0.015 | 58.0 |
|  | Blood | 0 | - | - | - | - |
|  | Missing | 0 | - | - | - | - |
|  | **Diagnose** |  |  |  |  |  |
|  | Liver biopsy | 8 | 1.03(1.00,1.07) | 0.047 | 0.615 | 0.0 |
|  | US | 2 | 2.49(1.35,4.61) | 0.004 | 0.109 | 61.2 |
|  | CT | 0 | - | - | - | - |
|  | **Measurement** |  |  |  |  |  |
|  | ELISA | 4 | 1.38(0.93,2.05) | 0.113 | 0.000 | 88.6 |
|  | Others | 6 | 1.04(1.01,1.08) | 0.014 | 0.934 | 0.0 |
|  |  |  |  |  |  |  |
| **MCP-1** | **Overall** | 6 | 1.04(0.96,1.12) | 0.337 | 0.064 | 52.1 |
|  | **Race** |  |  |  |  |  |
|  | Asian | 0 | - | - | - | - |
|  | Caucasian | 6 | 1.04(0.96,1.12) | 0.337 | 0.064 | 52.1 |
|  | Others | 0 | - | - | - | - |
|  | **Mean age** |  |  |  |  |  |
|  | ＜18 | 1 | 0.99(0.86,1.13) | 0.884 | - | - |
|  | 18-60 | 5 | 1.05(0.96,1.16) | 0.288 | 0.039 | 60.3 |
|  | ≥60 | 0 | - | - | - | - |
|  | Missing | 0 | - | - | - | - |
|  | **BMI** |  |  |  |  |  |
|  | ＜30 | 2 | 1.58(0.98,2.57) | 0.062 | 0.480 | 0.0 |
|  | ≥30 | 3 | 1.04(0.95,1.13) | 0.428 | 0.038 | 69.5 |
|  | Missing | 1 | 0.99(0.86,1.13) | 0.884 | - | - |
|  | **Sample** |  |  |  |  |  |
|  | Serum | 2 | 1.58(0.98,2.57) | 0.062 | 0.480 | 0.0 |
|  | Plasma | 4 | 1.03(0.96,1.10) | 0.450 | 0.077 | 56.3 |
|  | Blood | 0 | - | - | - | - |
|  | Missing | 0 | - | - | - | - |
|  | **Diagnose** |  |  |  |  |  |
|  | Liver biopsy | 4 | 1.03(0.96,1.10) | 0.450 | 0.077 | 56.3 |
|  | US | 0 | - | - | - | - |
|  | CT | 2 | 1.58(0.98,2.57) | 0.062 | 0.480 | 0.0 |
|  | **Measurement** |  |  |  |  |  |
|  | ELISA | 3 | 1.20(0.82,1.76) | 0.342 | 0.144 | 48.3 |
|  | Others | 3 | 1.04(0.95,1.13) | 0.428 | 0.038 | 69.5 |
|  |  |  |  |  |  |  |
| **IFN-γ** | **Overall** | 4 | 0.99(0.95,1.04) | 0.709 | 0.777 | 0.0 |
|  | **Race** |  |  |  |  |  |
|  | Asian | 0 | - | - | - | - |
|  | Caucasian | 4 | 0.99(0.95,1.04) | 0.709 | 0.777 | 0.0 |
|  | Others | 0 | - | - | - | - |
|  | **Mean age** |  |  |  |  |  |
|  | ＜18 | 1 | 1.03(0.90,1.18) | 0.669 | - | - |
|  | 18-60 | 3 | 0.99(0.94,1.03) | 0.587 | 0.683 | 0.0 |
|  | ≥60 | 0 | - | - | - | - |
|  | Missing | 0 | - | - | - | - |
|  | **BMI** |  |  |  |  |  |
|  | ＜30 | 0 | - | - | - | - |
|  | ≥30 | 3 | 0.99(0.94,1.03) | 0.587 | 0.683 | 0.0 |
|  | Missing | 1 | 1.03(0.90,1.18) | 0.669 | - | - |
|  | **Sample** |  |  |  |  |  |
|  | Serum | 0 | - | - | - | - |
|  | Plasma | 4 | 0.99(0.95,1.04) | 0.709 | 0.777 | 0.0 |
|  | Blood | 0 | - | - | - | - |
|  | Missing | 0 | - | - | - | - |
|  | **Diagnose** |  |  |  |  |  |
|  | Liver biopsy | 4 | 0.99(0.95,1.04) | 0.709 | 0.777 | 0.0 |
|  | US | 0 | - | - | - | - |
|  | CT | 0 | - | - | - | - |
|  | **Measurement** |  |  |  |  |  |
|  | ELISA | 1 | 1.03(0.90,1.18) | 0.669 | - | - |
|  | Others | 3 | 0.99(0.94,1.03) | 0.587 | 0.683 | 0.0 |
|  |  |  |  |  |  |  |
| **IGF-II** | **Overall** | 4 | 0.97(0.81,1.15) | 0.692 | 0.000 | 91.2 |
|  | **Race** |  |  |  |  |  |
|  | Asian | 0 | - | - | - | - |
|  | Caucasian | 4 | 0.97(0.81,1.15) | 0.692 | 0.000 | 91.2 |
|  | Others | 0 | - | - | - | - |
|  | **Mean age** |  |  |  |  |  |
|  | ＜18 | 1 | 1.05(0.92,1.20) | 0.485 | - | - |
|  | 18-60 | 3 | 0.94(0.75,1.18) | 0.588 | 0.000 | 93.8 |
|  | ≥60 | 0 | - | - | - | - |
|  | Missing | 0 | - | - | - | - |
|  | **BMI** |  |  |  |  |  |
|  | ＜30 | 0 | - | - | - | - |
|  | ≥30 | 3 | 0.94(0.75,1.18) | 0.588 | 0.000 | 93.8 |
|  | Missing | 1 | 1.05(0.92,1.20) | 0.485 | - | - |
|  | **Sample** |  |  |  |  |  |
|  | Serum | 0 | - | - | - | - |
|  | Plasma | 4 | 0.97(0.81,1.15) | 0.692 | 0.000 | 91.2 |
|  | Blood | 0 | - | - | - | - |
|  | Missing | 0 | - | - | - | - |
|  | **Diagnose** |  |  |  |  |  |
|  | Liver biopsy | 4 | 0.97(0.81,1.15) | 0.692 | 0.000 | 91.2 |
|  | US | 0 | - | - | - | - |
|  | CT | 0 | - | - | - | - |
|  | **Measurement** |  |  |  |  |  |
|  | ELISA | 1 | 1.05(0.92,1.20) | 0.485 | - | - |
|  | Others | 3 | 0.94(0.75,1.18) | 0.588 | 0.000 | 93.8 |
|  |  |  |  |  |  |  |
| **ICAM-1** | **Overall** | 4 | 2.17(1.22,3.85) | 0.008 | 0.000 | 88.2 |
|  | **Race** |  |  |  |  |  |
|  | Asian | 0 | - | - | - | - |
|  | Caucasian | 4 | 2.17(1.22,3.85) | 0.008 | 0.000 | 88.2 |
|  | Others | 0 | - | - | - | - |
|  | **Mean age** |  |  |  |  |  |
|  | ＜18 | 0 | - | - | - | - |
|  | 18-60 | 3 | 3.16(1.40,7.14) | 0.006 | 0.017 | 75.4 |
|  | ≥60 | 1 | 1.11(0.97,1.28) | 0.144 | - | - |
|  | Missing | 0 | - | - | - | - |
|  | **BMI** |  |  |  |  |  |
|  | ＜30 | 3 | 3.16(1.40,7.14) | 0.006 | 0.017 | 75.4 |
|  | ≥30 | 1 | 1.11(0.97,1.28) | 0.144 | - | - |
|  | Missing | 0 | - | - | - | - |
|  | **Sample** |  |  |  |  |  |
|  | Serum | 4 | 2.17(1.22,3.85) | 0.008 | 0.000 | 88.2 |
|  | Plasma | 0 | - | - | - | - |
|  | Blood | 0 | - | - | - | - |
|  | Missing | 0 | - | - | - | - |
|  | **Diagnose** |  |  |  |  |  |
|  | Liver biopsy | 0 | - | - | - | - |
|  | US | 1 | 1.80(1.35,2.41) | 0.000 | - | - |
|  | CT | 3 | 2.80(0.86,9.12) | 0.087 | 0.000 | 89.4 |
|  | **Measurement** |  |  |  |  |  |
|  | ELISA | 4 | 2.17(1.22,3.85) | 0.008 | 0.000 | 88.2 |
|  | Others | 0 | - | - | - | - |

Abbreviation: No. of studies, number of studies; OR (95%CI), odds ratios (95% confidence intervals); US, ultrasonography examination; CT, computed tomography.

**Supplementary Table 7.** Characteristics of studies assessing inflammasome in NAFLD.

| **Study (Year)** | **Reference** | **Design type** | **Inflammasome components** | **Results** |
| --- | --- | --- | --- | --- |
| Mehta (2012) | (52) | Cross-sectional | ASC/Caspase-1/IL-1β | mRNA levels of ASC were significantly higher in NAFLD patients |
| Schlattjan (2014) | (53) | Cohort | NLRP3 | Hepatic mRNA levels of NLRP3 were significantly correlated with NAFLD activity score |
| Adams (2015) | (54) | Cohort | NLRP3/Caspase-1 | Genetic polymorphisms in the NLRP3 inflammasome associated with altered serum caspase-1 and risk of NAFLD |
| Li (2015) | (55) | Case-control | NLRP3 /IL-1β/IL-18 | Level of IL-18 is significantly higher in NAFLD patients |
| Mitsuyoshi (2015) | (56) | Cross-sectional | NLRP3/ASC/Pro-caspase-1/IL-1β/IL-18 | Hepatic mRNA levels of NLRP3, ASC, pro-caspase- 1, IL-1β, and IL-18 were significantly higher in NAFLD patients;  Blood mRNA levels of pro-caspase-1 were significantly higher in NAFLD patients |
| Sowa (2015) | (57) | Cross-sectional | NLRP | Hepatic mRNA levels of NLRP were significantly correlated with NAFLD activity score |
| Kang (2016) | (58) | Cross-sectional | NLRP3/ NLRP6/ NLRP10 | Hepatic mRNA levels of NLRP3, NLRP6, NLRP10 were significantly higher in NAFLD patients;  Serum NLRP3 protein expression was significantly elevated in NAFLD patients |
| Neuman (2016) | (59) | Cohort | IL-1β/Caspase-3/Caspase-9 | Level of IL-1β is significantly higher in NASH patients |
| Mitsuyoshi (2017) | (60) | Cross-sectional | NLRP3 | Hepatic mRNA levels of NLRP3, procaspase- 1, IL-1β, and IL-18 were significantly higher in NAFLD patients;  Blood mRNA levels of pro-caspase-1 were significantly higher in NAFLD patients |
| Li (2019) | (61) | Case-control | NLRP3 /IL-1β/IL-18 | Level of IL-1β is significantly higher in NAFLD patients |
| Rau (2019) | (62) | Cohort | NLRP3 | Genetic polymorphisms in the NLRP3 inflammasome associated with serum ALT activities |
| Cyr (2020) | (63) | Case-control | NLRP3/ASC/IL-18 | Serum ASC and IL‐18 were significantly elevated in NASH patients |
| Sim (2020) | (64) | Case-control | Caspase-1/IL-18 | log10 IL-18 was significantly associated with AST and ALT |
| Unamuno (2020) | (65) | Case-control | NLRP3 /IL-1β/IL-18 | Hepatic mRNA levels of NLRP3, IL-1β, and IL-18 were significantly higher in NAFLD patients |
| Quezada (2021) | (66) | Cross-sectional | NLRP3/IL-1β | IR and liver histopathology were correlated with NLRP3 inflammasome components as well as with liver and plasma IL-1β levels |
| Unamuno (2020) | (67) | Case-control | NLRP3/NLRP6/IL-1β/NLRP1/IL-18 | NLRP3 in visceral adipose tissue was significantly elevated in NASH;  mRNA levels of NLRP6, IL-1β, and ASC in visceral adipose tissue were significantly higher in NAFLD patients;  mRNA levels of NLRP1 and IL18 were elevated in patients with liver disease |
| Sim (2021) | (68) | Cross-sectional | Caspase-1/IL-18 | IL-18 was significantly associated with AST and ALT |
| Unamuno (2021) | (69) | Case-control | NLRP3 | Hepatic mRNA levels of NLRP3, IL-1β, and IL-18 were significantly higher in NAFLD associated comorbidities |
| Zhu (2021) | (70) | Case-control | NLRP3/Caspase-1/ IL-1β/IL-18 | mRNA levels of NLRP3, caspase-1, IL-1β, and IL-18 were significantly elevated in peripheral blood mononuclear cells of NAFLD patients |

Abbreviation: NLRP, NOD-like receptor protein; ASC, apoptosis-associated speck-like protein containing a CARD; IL-1β, interleukin-1β; IL-18, interleukin-18.
